# Genome Assembly of the Fungus *Cochliobolus miyabeanus*, and Transcriptome Analysis during Early Stages of Infection on American Wildrice (*Zizania palustris* L.)

**DOI:** 10.1371/journal.pone.0154122

**Published:** 2016-06-02

**Authors:** Claudia V. Castell-Miller, Juan J. Gutierrez-Gonzalez, Zheng Jin Tu, Kathryn E. Bushley, Matthieu Hainaut, Bernard Henrissat, Deborah A. Samac

**Affiliations:** 1 Department of Plant Pathology, University of Minnesota, Saint Paul, Minnesota, United States of America; 2 Department of Agronomy and Plant Genetics, University of Minnesota, Saint Paul, Minnesota, United States of America; 3 USDA-ARS-Plant Science Research Unit, Saint Paul, Minnesota, United States of America; 4 Mayo Clinic, Division of Biomedical Statistics and Informatics, Rochester, Minnesota, United States of America; 5 Department of Plant Biology, University of Minnesota, Saint Paul, Minnesota, United States of America; 6 CNRS UMR 7257, Aix-Marseille University, Marseille, France; 7 INRA, USC 1408 AFMB, Marseille, France; 8 Department of Biological Sciences, King Abdulaziz University, Jeddah, Saudi Arabia; The University of Wisconsin - Madison, UNITED STATES

## Abstract

The fungus *Cochliobolus miyabeanus* causes severe leaf spot disease on rice (*Oryza sativa*) and two North American specialty crops, American wildrice (*Zizania palustris*) and switchgrass (*Panicum virgatum*). Despite the importance of *C*. *miyabeanus* as a disease-causing agent in wildrice, little is known about either the mechanisms of pathogenicity or host defense responses. To start bridging these gaps, the genome of *C*. *miyabeanus* strain TG12bL2 was shotgun sequenced using Illumina technology. The genome assembly consists of 31.79 Mbp in 2,378 scaffolds with an N_50_ = 74,921. It contains 11,000 predicted genes of which 94.5% were annotated. Approximately 10% of total gene number is expected to be secreted. The *C*. *miyabeanus* genome is rich in carbohydrate active enzymes, and harbors 187 small secreted peptides (SSPs) and some fungal effector homologs. Detoxification systems were represented by a variety of enzymes that could offer protection against plant defense compounds. The non-ribosomal peptide synthetases and polyketide synthases (PKS) present were common to other *Cochliobolus* species. Additionally, the fungal transcriptome was analyzed at 48 hours after inoculation *in planta*. A total of 10,674 genes were found to be expressed, some of which are known to be involved in pathogenicity or response to host defenses including hydrophobins, cutinase, cell wall degrading enzymes, enzymes related to reactive oxygen species scavenging, PKS, detoxification systems, SSPs, and a known fungal effector. This work will facilitate future research on *C*. *miyabeanus* pathogen-associated molecular patterns and effectors, and in the identification of their corresponding wildrice defense mechanisms.

## Introduction

*Cochliobolus miyabeanus* ((Ito & Kuribayashi) Drechsler ex Datur.) (anamorph = *Bipolaris oryzae* (Breda de Haan) Shoemaker) is a common fungal pathogen worldwide. In the U.S., it has been documented from the north, in North Dakota and Minnesota, to the south, from Florida to Texas, as well as in areas of California [1–4]. It is a major pathogen of rice (*Oryza sativa* L.) in all areas of the world where this crop is grown [5]. In addition, it has the potential to cause a severe yield limiting leaf spot disease on two North American non-traditional grass crops, switchgrass (*Panicum virgatum* L.), grown for cellulosic biofuel production [4], and American wildrice (*Zizania palustris* L.), hypothesized to have originated in North America [6] and grown commercially for its gourmet grain [7]. *C*. *miyabeanus* causes fungal brown spot (FBS) on wildrice that can lead to economically disastrous losses in paddy-grown crops [8, 9], resulting in a greater reliance on fungicide to bring about profitable yields. In susceptible wildrice, fungal conidia usually germinate by 8 h after deposition on leaves and develop club-shaped appressoria by 18 h. Infection hyphae break through the cuticle, or less frequently through stomata, develop under the cuticle, and later invade inter- and intracellular spaces. Symptoms appear about 18 to 48 h after inoculation as brown-purple to dark spots that enlarge over time into oval lesions with brown to tan necrotic centers, frequently surrounded by chlorotic halos [10]. Lesions tend to coalescence, whitening aerial leaves. Stems and sheaths can also be infected and the weakened stems frequently break, causing considerable kernel loss [3].

To mitigate grain yield reduction, a few wildrice cultivars have been released with improved genetic resistance to FBS [11]; however, the molecular bases of resistance are not known. Further, fungal mechanisms of virulence on wildrice have not been broadly studied in contrast to those of other species of *Cochliobolus*.

Pathogenicity in many *Cochliobolus* species is largely due to host-specific toxins (HSTs). The first HST described was victorin, a nonribosomal peptide (NRP), produced by *C*. *victoriae*, the causal agent of Victoria blight in oat [12]. It has been proposed that in susceptible oat genotypes carrying the homozygous dominant *Vb* locus, the fungal toxin binds to a thioredoxin guarded by a NB-LRR protein that in turn triggers apoptosis, facilitating disease for this necrotrophic fungus [13]. *C*. *carbonum* race 1 produces HC-toxin, a tetrapeptide that inhibits histone deacetylases involved in DNA repair, modification, and transcription. The locus *TOX2* contains essential genes for toxin synthesis which includes *HTS1*, a nonribosomal peptide synthetase (*NRPS*) [14], *TOXA*, a cyclic peptide efflux pump for toxin detoxification [15], *TOXC*, a fatty acid synthetase [16], *TOXF*, a branched-chain amino acid transaminase [17], *TOXG*, an alanine racemase [18], and *TOXE*, an atypical regulatory sequence that controls expression of *TOXA* and *TOXC* [19]. Race T of *C*. *heterostrophus* produces a linear polyketide HST (T-toxin), which generates pores in the inner mitochondrial membrane and subsequent leakage in maize carrying the Texas male sterile cytoplasm (*T-urf13*) gene. The complex locus *TOX1* includes genes for synthesis of the toxin found in two unlinked loci, *ToxA* and *ToxB* [20]. *ToxA* contains two monomodular polyketide synthase (*PKS*) genes required for toxin production and virulence [21, 22]. *ToxB* comprises a decarboxylase (*DEC1*) and three reductases (*RED1*, *RED2*, and *RED3*) [20, 23] of which only *RED2* participates in the toxic peptide synthesis. Lastly, a putative NRPS associated with virulence on barley was recently uncovered in *C*. *sativus* through comparative genomics among *Cochliobolus* species [24].

*C*. *miyabeanus* strains that are pathogenic on common rice do not have unique PKSs or NRPSs and are not known to produce an HST [24]. *C*. *miyabeanus*, and other *Cochliobolus* species, make non-specific phytotoxic cyclic sesquiterpenes commonly known as ophiobolins [25], which are also produced by non-pathogenic fungi [26], suggesting that they have functions other than in interactions with plant hosts. Purified ophiobolins have been found to have a number of effects on plants including inhibiting root growth, stimulating electrolyte leakage from roots, and inducing stomatal opening [26]. Ophiobolins also have antimicrobial activity and cause hyphal deformation [27].

*Cochliobolus* belongs to the class Dothidiomycetes. This class consists of fungi with a wide assortment of life styles that live in ecologically diverse environments. It is thought that members of this class descended from a common ancestor over 280 million years ago, and contemporary species exhibit genomes with macro-, meso-, and microsynteny, variation in genome sizes attributed to the amount of repeated DNA, and yet conserved gene numbers [28]. Plant pathogens within the Dothidiomycete*s* contain genes with 10 unique Pfam domains and 69 expanded domains that are not present in other plant pathogens. The proteomes of plant pathogens within the *Pleosporales* are enriched for cysteine-rich small secreted proteins (SSPs) ≤ 200 amino acids (aa) in length, some of which are thought to be involved in plant-fungus interactions. Some SSPs are common to all *Cochliobolus* species while others are unique to single species [24]. The Dothideomycetes have a vast number of genes for production of secondary metabolites, including PKSs, NPSs, and terpene synthases [28]. A comparative study of *Cochliobolus* plant pathogens showed that some of the secondary metabolites synthesized by *NRPS* and *PKS* genes were conserved among *Cochliobolus* species, while others were unique to a single species. A few of them (NPS1, NPS3, and NPS13) with discontinuous distribution among species have complicated patterns of evolution that include expansion, loss, and recombination of adenylation (AMP) domains that may generate novel plant toxic peptides [24].

Here, we report the draft genome assembly and catalog of genes of a *C*. *miyabeanus* strain originally isolated from infected wildrice as well as the transcriptome of the pathogen during an early time point of wildrice colonization. In our analysis we focus on potential effectors, including small secreted proteins and genes potentially involved in pathogenicity on wildrice, with the objective of gaining a better understanding of the mechanisms of pathogenesis to assist in enhancing genetic resistance in wildrice breeding lines.

## Materials and Methods

### Fungal strain and DNA extraction

The *C*. *miyabeanus* strain TG12bL2 (hereafter referred to as *Cm*TG12bL2) was isolated from a wildrice leaf with FBS symptoms collected from a paddy in Aitkin, Minnesota, USA as previously described [29]. For DNA extraction, the fungus was grown in 2% (w/v) water agar (Bacto Agar, DIFCO) for approximately two weeks until spores were produced. Spores collected from four Petri dishes were added to two sterilized 500 ml glass flasks containing 200 ml of liquid minimal medium [30]. The flasks were shaken at 150 rpm for 6 days at room temperature in ambient light, breaking the mycelium apart every 48 h to promote new growth, and placing in fresh medium. Mycelium was harvested by filtration, freeze dried, and DNA was extracted using the protocol of Raeder and Broda [31]. DNA concentration and quality were measured using the Agilent 2100 Bioanalyzer (Agilent Technologies, Palo Alto, CA).

### Fungal genome sequencing and *de novo* assembly

The *Cm*TG12bL2 genome was shotgun sequenced using paired-end reads with 101 cycles with Illumina HiSeq 2000 technology at the Mayo Clinic, Rochester, Minnesota, USA. A paired-end library was prepared according to the Illumina Preparation Guide (http://www.illumina.com). Briefly, 2 to 5 μg of DNA in 100 μl of 10 mM Tris and 0.1 mM EDTA, pH 8, was fragmented using a Covaris E210 sonicator (Woburn, MA) generating double-stranded DNA fragments with blunt or sticky ends with a fragment size mode between 400 to 500 bp. The ends were repaired using Klenow DNA polymerase and T4 DNA polymerase, phosphorylated using T4 polynucleotide kinase, after which an adenine was added to the 3’ ends of double-stranded DNA using Exo-Minus Klenow DNA polymerase. Paired-end DNA adaptors with a single thymine base overhang at the 3’ end were ligated to DNA fragments and the resulting constructs were separated on a 2% agarose gel. DNA fragments of approximately 500 bp were excised from the gel using GeneCatcher tips and purified using Qiagen Gel Extraction Kits (Qiagen, Valencia, CA). The adapter-modified DNA fragments were enriched by 12 cycles of PCR using primers PE 1.0 and PE 2.0. The concentration and size distribution of the library was determined with a DNA 1000 chip on the Agilent 2100 Bioanalyzer (Agilent Technologies). The library was loaded onto an indexed lane of paired-end flow cell. The reads in the flow cell were sequenced as paired-end indexed reads on an Illumina HiSeq 2000 using TruSeq SBS sequencing kit version 1 and HiSeq data collection v. 1.1.37.0 software. Base-calling was performed using Illumina’s RTA v. 1.7.45.0. Reads quality control was performed in Galaxy (https://galaxyproject.org/).

The fungal genome was *de novo* assembled using Velvet v.1.2.10 [32]. Hash length (*k*-mer) ranged from 31 to 91 by two nucleotide increments. Contigs were merged into scaffolds using paired-end information and a coverage cutoff and expected coverage set to ‘auto’ and an average insert length of 385 bp. The optimal hash length for the assembly was selected based on the maximum N_50_ length, large *k*-mer specificity, and high coverage, without losing overall genome or scaffold length. Parameter optimization for the selected assembly was further refined using the expected coverage (76.56×) and cutoff coverage (38.28×), following software recommendations [33], to discriminate unique genomic regions from repeats, and to further eliminate errors due to low coverage, respectively.

### Protein-coding sequence prediction, annotation, and validation

Protein-coding sequences were predicted using GeneMark_ES v.2.3c [34], which employs an intrinsic method with unsupervised training based on an *ab initio* algorithm. The algorithm features an enhanced intron sub-model to accommodate sequences with and without branch point sites found in several fungi (Ascomycota, Basidiomycota, and Zygomycota). Proteins were annotated against NCBI non-redundant (NCBI_nr) (http://www/ncbi.nlm.nih.gov) and HMMER (http://hmmer.janelia.org) databases. The latter was also used to identify protein profiles (Pfam). For overall analyses with blastp an *e*-value cut off of 1E-5 was used, except for detecting candidate effectors where a higher *e*-value cut off was permitted (1E-2). Proteins with a signal peptide or anchor peptide were predicted by SignalP 4.1 [35]. Small secreted proteins (SSPs) were considered those of less than 200 aa without transmembrane domains. Cysteine-rich SSPs in the *Cm*TG12bL2 genome were identified as previously described [28]. HCSSPs were resolved as in [28] with at least twice the average of cysteines in the *Cm*TG12bL2 proteome and as in [24] as those SSPs containing more than 2% cysteines.

The *Cm*TG12bL2 predicted genes were validated using three strategies. First, the protein set was compared to a core of 458 highly conserved proteins [36]. This core consisted of highly homologous proteins that are present in six eukaryote genomes (*Homo sapiens*, *Drosophila melanogaster*, *Caenorhabditis elegans*, *Arabidopsis thaliana*, *Saccharomyces cerevisiae*, and *Schizosaccharomyces pombe*), and was selected from the KOG (clusters of euKaryotic Orthologous Groups) database. Second, the genomes of *Cm*TG12bL2 and *C*. *miyabeanus* WK-1C (ATCC 44560 v1.0) (http://genome.jgi.doe.gov) were compared using Gepard [37]. Third, by comparing the genes to the fungal transcriptome at 48 h post-inoculation that was assembled and identified as described below. We adopted the following nomenclature throughout the manuscript: “Cm” refers to gene; “CM” to protein and “t_Cm” to transcript. The number that follows each prefix is the same for gene, protein, and transcript.

### Identifying carbohydrate-active enzymes (CAZymes)

For the detection of encoded CAZymes, each *Cm*TG12bL2 protein model was compared (blastp) to proteins listed in the Carbohydrate-Active Enzymes database (www.cazy.org) [38]. Model *Cm*TG12bL2 proteins with a length of over 50% identity to those in CAZy database were directly assigned to the same family (or subfamily when relevant). Proteins with less than 50% identity to a protein in CAZy were all manually inspected for conserved features such as the catalytic residues. Sequence alignments with isolated functional domains were performed in the case of multimodular CAZymes [39]. The same methods were used for all fungi that were compared to *Cm*TG12bL2 proteins. Putative roles of the enzymes were inferred from annotations of proteins returned from searches against the profile-HMMER database (hmmscan) and protein sequences database (phmmer) using an *e*-value cut off of 1E-33.

### Nonribosomal peptide synthetase and polyketide synthase identification and phylogenetic analysis

NRPSs and PKSs were identified by comparing the *Cm*TG12bL2 proteins to the well-curated dataset of *C*. *heterostrophus* (Ch_NPSs) [40, 41] using an *in-house* script and to other NRPSs and PKSs from NCBI_nr (blastp, 1E-5). Additional motifs in proteins with AMP domains and ketoacyl synthase (KS) domains were identified using HMMER hmmscan (with default settings unless otherwise specified). A degenerate AMP domain in the predicted *Cm*TG12bL2 protein CM_1661, homologous with Ch_NPS6 was identified through manual inspection [42]. AMP domains of CM_3163 were compared to the corresponding *C*. *heterostrophus* homologous domains NPS1, NPS3, and NPS13. Finally, putative NRPSs and PKSs from *Cm*TG12bL2 were blasted to proteins of *C*. *miyabeanus* WK-1C. A phylogenetic analysis with the *Cm*TG12bL2 AMP domains was done using methods described by Bushley and Turgeon [40] to compare conservation and relationships of AMP domains with those identified in the reference species *C*. *heterostrophus* C5, *C*. *heterostrophus* C4, and the rice isolate *C*. *miyabeanus* WK-1C [24]. Several AMP domains from NRPSs in other Ascomycetes species that encode known chemical products were included as well. Acyl-adenylating enzymes (acyl-CoA-synthetases, *CPS1*, long chain fatty acids, acyl-CoA-ligases, and ochratoxins) from *C*. *heterostrophus* and other ascomycetes were used as outgroups. Protein sequences were aligned using MAFFT and a maximum likelihood phylogenetic tree was constructed in RAxML using the best protein model determined by ProTest (RT-REV-F) as in [24, 40].

### Predicted transporter and detoxification systems

P450 monooxygenases were obtained by comparing the *Cm*TG12bL2 protein set (blastp, *e*-value cut off 1E-5) to the Fungal Cytochrome P450 Database (FCPD; http://p450.riceblast.snu.ac.kr/download.php). The MFS and ABC transporters were predicted by comparison to the transporter classification database (www.tcdb.org) with an *e*-value cut off of 1E-05.

## Wildrice inoculation with *C*. *miyabeanus*

Leaves of wildrice plants of the variety Itasca C-12 with improved resistance to FBS [11] were inoculated with 20,000 conidia/ml of the isolate *Cm*TG12bL2, in four biological replicates. Plants were kept in a mist chamber for 16 h then moved to a greenhouse and kept at 24°C with 75% relative humidity. Samples of the flag leaf and flag leaf-1 were collected at 24 h and 48 h after inoculation (hai), immediately frozen in liquid nitrogen, and kept at -80°C until used for RNA extraction.

### *C*. *miyabeanus* transcriptome assembly and analysis

Total RNA was extracted with the RNeasy Mini Kit (Qiagen Inc, Valencia, CA) according to manufacturer’s instructions and treated with Ambion DNAse I (Life Technologies, Carlsbad, CA) to eliminate traces of genomic DNA. Only samples collected at 48 hai were sequenced for the transcriptome analysis. The Illumina TruSeq RNA Sample Preparation Guide was followed to prepare the samples for sequencing according to manufacturer’s recommendations (http://www.illumina.com). Illumina library preparation was performed as previously described [43]. The samples were barcoded, multiplexed, and sequenced using a single-end read with 50 cycles using an Illumina HiSeq2000 machine at the Biomedical Genomic Center at the University of Minnesota. Read quality control was carried out in Galaxy using the Tuxedo suite. Only reads with an average Q score ≥30 were aligned onto the *Cm*TG12bL2 draft genome for transcriptome assembly using TopHat v1.4.1 [44] implemented in Galaxy.

The CuffLinks suite of tools [45] was used for transcript assembly and quantification. Transcript abundance was estimated using RPKM (Reads Per Kilobase of transcript per Million mapped reads) [46]. RPKM values were log_2_ transformed and data distribution was visualized using JMPin (SAS Institute Inc., Cary, NC, USA). Experimental variability of the log_2_ of RPKM between biological replicates was estimated using Pearson’s correlation coefficient for the 9,960 transcripts that were present in all replicates. For subsequent data analysis only transcripts found in at least three of four replicates were used. Transcripts were functionally categorized using Blast2GO (www.blast2go.com) against the NCBI non-redundant (NCBI_nr) and SwissProt/InterPro databases. An enrichment analysis was performed with the 10% most abundant transcripts using a Fisher exact test with a false discovery rate (FDR) of 0.05 implemented within Blast2GO [47] to identify overrepresented or enriched GO terms using the whole set of transcripts as reference.

### Quantitative RT-PCR (qRT-PCR) experiments

qRT-PCR experiments were done to validate expression of the transcriptome analysis at 48 hai and to compare the expression levels to an additional time point at 24 hai *in planta* and to the fungus grown *in vitro*. cDNA synthesis for all the treatments was carried out with the iScript cDNA synthesis kit (Bio-Rad Laboratories Inc., Hercules, CA). *Cm*TG12bL2 specific primers for the selected genes *Ecp6* (Cm_2799), CYP53 (Cm_8068), salicylate hydroxylase (Cm_9653), β-1,4-endoglucanase (Cm_1858), β-1,4-endoxylanase (Cm_8238), and β-1,4-glucosidase (Cm_28)) were designed using the SciTools software at IDT (http://www.idtdna.com/scitools/Applications/RealTimePCR). The genes expression levels *in planta* were compared to those of the fungus grown *in vitro* (reference sample). qRT-PCR reactions were done in 96-wells plates using an Applied Biosystems 7500/7500 Fast Real-Time PCR system (Applied Biosystems, Foster City, CA). Each reaction of 20 μL contained 10 μL iTAQ^™^ Universal SYBR Green Supermix (Bio-Rad Laboratories Inc.), 0.1 μL of each primer diluted to 0.1μM, 3 μL of cDNA template and 6.8 μL of nuclease-free water. PCR condition were 95°C for 2 min, 40 cycles of 95°C for 3 s, 60°C for 30 s, and 95°C for 15 s, followed by 60°C for 1 min, 95°C for 15 s, 60°C for 15 s (melt curve generation). The delta-delta Ct method was computed by the Applied Biosystems software and used for relative quantification of gene expression. Expression data were normalized against an endogenous control (glyceraldehyde-3-phosphate dehydrogenase gene). Two to three biological replications with at least two technical replicates were analyzed per gene. Primer efficiencies for the genes analyzed were 100% ±10 (SD). Data are presented as the Log_2_ of relative gene expression (fold change). Expression values in the reference sample were set to 0 (Log_2_ of 1). Significance of gene differential expression between treatments was tested using T-tests with the significance threshold set at 0.05.

## Results

### *C*. *miyabeanus* fungal genome assembly, gene prediction, and validation

Read statistics and quality are summarized in S1 Table. High quality reads were assembled with various *k*-mer lengths (S1 Fig). The *65*-mer assembly yielded the largest N_50_ (75,371 bp), and contained 3,475 scaffolds, with a maximum scaffold size of 348,044 bp and a total genome length of 31,696,836 bp (S1 Fig). After parameter optimization (S2 Fig) and removal of contigs shorter than 100 bp, the final draft was 31,788,735 bp long (Table 1) and contained 2,378 scaffolds with a N_50_ value of 74,921 bp. This Whole Genome Shotgun project has been deposited at DDBJ/EMBL/GenBank under the accession LNFW00000000. The version described in this paper is version LNFW01000000.

**Table 1:** Summary features of the *Cochliobolus miyabeanus Cm*TG12bL2 isolate genome assembly and annotation.

Parameter	
Genome size (draft) (bp)^a^	31,788,735
Number of scaffolds	2,378
Mean/Maximum scaffold length (bp)	13,368/348,108
Scaffold N_50_ length (bp)	74,921
Number of scaffolds with length over N_50_	131
Mean coverage (X)	229.30
GC genome content (%)	50.50
Total predicted protein-coding genes^b^	12,142
Partial genes^c^	1,142
Predicted complete set of protein-coding genes^d^	11,000
Mean coding gene length (bp)^e^	1,588
Mean exon length (bp)^e^	529
Mean intron length^e^	93
Mean number of exon/gene^e^	2.71
Mean gene density^f^ (genes/Mbp)	346
Mean amino acid number/protein	476
Genes with a signal peptide^g^	1,066

^a^ Genome size: total base pairs after optimizing the parameters for cutoff coverage (38.3X), expected coverage (76.56X), and removing contigs smaller than 100 bp.

^b^ Total number of protein-coding genes as predicted by GeneMark-ES v.2.3c.

^c^ Predicted gene sequences lacking part of the coding region (i.e. start or stop codons) or with stop codon(s) in the sequences.

^d^ Total number of predicted complete genes used in further analyses.

^e^ Mean values based on 11,000 genes.

^f^ Calculated as total protein coding gene sequences/ (protein-coding gene sequences + total intergenic areas).

^g^ Predicted using SignalP4.1 server and based on 11,000 genes.

The total number of protein-coding sequences initially predicted by GeneMark-ES was 12,142. After discarding partial genes (1,142), due to the presence of internal stop codons or lack of either initiation or stop codons, 11,000 coding sequences remained for further analyses. The average C+G content was 52.63% for predicted genes, 52.74% for exons, and 45.69% for introns, *versus* 50.5% for the entire genome. Additional genome assembly features are shown in Table 1. Interrogation (blastp, 1E-05) of the 11,000 predicted proteins with the NCBI_nr database resulted in 10,395 matches (94.5%), of which 92.3% were annotated as hypothetical proteins, mostly belonging to other *Cochliobolus* species (92.2%). A total of 7,567 motifs were identified when comparing (blastp, 1E-05) all predicted proteins to collections of protein profiles (http://hmmer.janelia.org/).

*Cm*TG12bL2 gene models were also validated (blastp, 1E-05) against a core dataset of 458 highly conserved eukaryote proteins [36]. All but two of the 458 core proteins had at least one predicted *Cm*TG12bL2 homolog (S2 Table). Of the two core proteins that could not be directly identified: KOG1291 (Mn^2+^ and Fe^2+^ transporters of the NRAMP family) and KOG2749 (mRNA cleavage and polyadenylation factor IA/II complex), the latter matched a partial protein. Additionally, two *Cm*TG12bL2 proteins, CM_8436 and CM_2839, each matched two core proteins, but only one of each *Cm*TG12bL2 protein (highest percentage of identity) is presented. Thus, 99.1% of the core dataset could be identified in our predicted protein set. The *Cm*TG12bL2 genome assembly was compared to the *C*. *miyabeanus* WK-1C genome (http://genome.jgi.doe.gov). The ordered scaffolds showed good pair-wise alignment (S3 Fig) validating the assembly over relatively long distances. An additional validation was done by mapping fungal transcripts against the draft genome. Overall, 97.6% of the predicted genes that were expressed at 48 hai in at least three biological replicates were mapped to the genome sequence.

### Genes involved in plant-pathogen interactions

Comparative genome analyses identified a number of genes in *Cm*TG12bL2 previously shown to be required for pathogenicity in other *Cochliobolus* species (Table 2). For instance, the *C*. *heterostrophus CGBI* gene encodes a signaling G-protein ß-subunit involved in conidia production and female fertility as well as appressorium and mycelium pigmentation, mycelium morphogenesis, and virulence [48]. The *Cm*TG12bL2 putative homolog has eight exons and seven introns and encodes a polypeptide with two conserved domains, a WD40 domain and a phosducin-like domain that is a cytosolic regulator of G-proteins from the thioredoxin-like superfamily. The *C*. *heterostrophus CPS1* gene encodes an acyl CoA ligase-like protein, which is required for normal virulence in maize. Because it is present in several pathogenic and saprophytic Ascomycetes *CPS1* may also have a role in stress tolerance [49]. The *Cm*TG12bL2 homolog contains two large AMP-binding domains, and a DMAP1-binding domain at its amino terminal end. The *HDC1* to *HDC4* genes of *C*. *carbonum* encode histone deacetylases [50, 51]. *HDC1* is required for virulence (penetration efficiency) in maize, normal conidia size, and growth in complex polysaccharides [50]. The *Cm*TG12bL2 homolog is identical to the 505 amino acid (aa) sequence of *HDC1* but is longer at the carboxyl terminus. An 880 aa protein of *Cm*TG12bL2 was 99% identical to the protein kinase ccSNF1 from *C*. *carbonum*, including the predicted nuclear localization signal [52]. ccSNF1 regulates expression of extracellular fungal enzymes for degradation and uptake of carbohydrates and is required for full virulence in maize. A homolog of the DNA-binding transcriptional repressor CreA [53] for fungal degrading enzymes of plant cell walls, and putatively regulated by ccSNF1 in *C*. *carbonum*, is also present in the *Cm*TG12bL2 genome. The *BKM1* gene of a rice isolate of *C*. *miyabeanus* has a homolog in *Cm*TG12bL2. Disruption of *BKM1* causes defective mycelia and colony growth, loss of conidiation and pathogenicity on rice leaves [54]. A homolog of the Ch_NPS6, required for virulence and insensitivity to hydrogen peroxide in rice [55] is also present in the *Cm*TG12bL2 genome.

**Table 2:** *Cochliobolus miyabeanus* TG12bL2 genes with similarity to genes involved plant-pathogen interactions in other *Cochliobolus* species.

*C*. *miyabeanus* gene ID	Gene name in other *Cochliobolus* species	*e*-value (Percent amino acid identity; percent of sequence coverage)	Function of homolog in *Cochliobolus* sp.	Reference
Cm_7805	SNF1 (AF159253)	0.0 (99; 100)	Catabolic-repression and uptake	[52]
Cm_6457	CGB1 (AAO25585)	0.0 (100; 66)	Signaling G-protein β-subunit	[48]
Cm_6993	BMK1 (BAD42855)	0.0 (100; 95)	Development pathways	[54]
Cm_5509	CreA (Q9HFS2)	0.0 (98; 100)	DNA binding transcriptional repressor	[53]
Cm_10788	CPS1 (AAF53991)	0.0 (96; 89)	Acyl-CoA ligase/stress response	[49]
Cm_6585	HDC1 (AF306507)	0.0 (100; 68)	Histone deacetylase	[50]
Cm_3336	HDC2 (AF349677)	0.0 (99; 100)	Histone deacetylase	[50]
Cm_3871	HDC3 (AF307341)	0.0 (98; 100)	Histone deacetylase	[51]
Cm_10259	HDC4 (AF537126)	0.0 (95; 99)	Histone deacetylase	[51]
Cm_1661	NPS6 (ABI51982)	0.0 (99; 91)	Iron extracellular siderophore	[55]
Cm_1096	CHAP1 (AAS64313)	0.0 (91; 100)	Redox-regulated transcription factor	[56]
Cm_3707	PGN1 (P26215)	0.0 (95; 100)	Endopolygalacturonase	[57]
Cm_30	PGX1 (Q00359)	0.0 (96; 100)	Exopolygalacturonase	[58]
Cm_3810	XYL2 (AAT49296)	0.0 (97; 100)	Endo-beta-1,4 xylanase	[59]
Cm_10775	XYL3 (AAC62816)	9E^-160^ (96; 100)	Endo-beta-1,4 xylanase	[59]
Cm_1858	CEL2 (AF336799)	0.0 (95, 100)	Cellulase	[60]
Cm_8632	ALP1 (AAB03851)	3E^-167^ (87; 100)	Trypsin-like serine protease	[61]

The *Cm*TG12bL2 genome has genes that encode homologs of proteins involved in causing disease but are not required for pathogenicity (Table 2). An example is a predicted homolog of *CHAP1* that in *C*. *heterostrophus* encodes a redox-regulated transcription factor necessary for gene activation for resistance to oxidative stress, although it is not involved in virulence in maize [56]. The *Cm*TG12bL2 genome has homolog proteins to five *C*. *carbonum* cell wall degrading enzymes, endopolygalacturonase (PGN1), exo-α-1,4-polygalacturonase (PGX1), two ß-1,4-xylanases (XYL2 and XYL3), and glucohydrolase (cellulase; CEL2), which were proven to be dispensable because strains with mutations in those genes were still pathogenic in maize [57, 58, 59, 60]. Additionally a *Cm*TG12bL2 protein was similar to *C*. *carbonum ALP1*, a trypsin-like serine protease that is not required for pathogenicity in maize [61]. Several of the *Cm*TG12bL2 genes are expected to have similar functions in the *C*. *miyabeanus*-wildrice interaction, particularly genes such as the iron extracellular siderophore *NPS6*, and *BKM1* that are involved in virulence/pathogenicity in the rice isolate of *C*. *miyabeanus* [54, 55].

### Small secreted proteins (SSPs)

The fungal secretome is essential for interactions with the surrounding environment and potential hosts. Within the SSP group are cysteine-rich secreted proteins (candidate effectors) that could interfere with plant defense mechanisms. The *Cm*TG12bL2 proteome includes a total of 187 proteins less than 200 aa long, with signal peptides and without transmembrane domains. The number of SSP proteins is comparable to the number found in other Dothidiomycetes, although less numerous than those found in other *Cochliobolus* species [28] (S3 Table). Most of the *Cm*TG12bL2 SSPs (95.7%) have similarities to hypothetical proteins from other *Cochliobolus* species in the NCBI_nr database including *C*. *miyabeanus* WK-1C. SSP features (S3 Table) are within or close to the range limits found in *Cochliobolus* species or other Dothidiomycetes [24, 28]. The percentage of SSPs with Pfam domains in *Cm*TG12bL2 (13.9%) is higher, albeit close to that found in other Dothidiomycetes. Examples of proteins with motifs identified in *Cm*TG12bL2 SSPs are hydrophobins, hydrophobic peptides with predicted functions as surfactants [62], CAZymes, essential to the hydrolysis of carbohydrates of plant cells walls, and proteins with carbohydrate-binding modules (described elsewhere in the manuscript). Other proteins included a fungal-specific CFEM protein with eight conserved cysteines, some of which have been implicated in fungal pathogenicity [63], a protein with an Hce2 domain that constitutes the mature part of the *Ecp*2 effector protein from the tomato pathogen *Cladosporium fulvum* (detailed in the next section), and a protein similar to Asp f 13-like from *C*. *lunatus* that contains a cerato-platanin domain. Proteins with this domain are phytotoxic and cause cell necrosis and induce plant defenses [64] but also have structural functions in the fungal cell wall. Additional SSPs identified included a clathrin adaptor complex small chain protein involved in the clathrin-mediated pathway for endocytosis of small molecules and a peptidase inhibitor I9 (S4 Table).

### Effector-like secreted proteins

Some of the effector-like proteins present in *Cm*TG12bL2 are similar to proteins known to be involved in pathogenicity, or to interfere with plant defense mechanisms. One example is CM_6804 with a pathogen effector Hce2 motif, a putative necrosis-inducing factor. This protein is similar to the extracellular protein 2 (Ecp2) of the biotrophic pathogen *Cladosporium fulvum* (S4 Fig), that is secreted into the apoplast during colonization of tomato leaves and is considered to have a role in virulence [65]. *Cm_6804* is 488 bp long, with two exons and one intron, and encodes a 142 aa peptide with a predicted signal peptide of 19 aa. Similar proteins have been found in *Mycosphaerella graminicola* and *M*. *fijiensis* [66], *Gibberella zeae*, *C*. *sativus* ND90Pr, and *C*. *heterostrophus* (S4 Fig) and they could play an important role in pathogenesis.

The CM_2799 protein is similar to *C*. *fulvum* Ecp6, which is secreted into the apoplast of tomato leaves during colonization and is involved in virulence [67]. Ecp6 is found in many other plant pathogenic and saprophyte fungi [67, 68]. The three Epc6 LysM domains act as carbohydrate binding modules with affinity to chitin tri-, penta-, and hexa-oligosaccharides and compete with chitin-binding plant receptors, thus preventing initiation of defense responses by the plant immune system [69]. CM_2799 has three typical LysM domains (pfam01476) with one cysteine at the beginning and end of each domain (S4 Fig), which could form intramolecular disulfide bonds. In addition, the protein has a cysteine at the C-terminus. Similar peptides are found in other fungi [69] including the hemibiotrophs *C*. *sativus* NDPr90, *Collectotrichum truncatum*, *Setosphaeria turcica*, and *Mycosphaerella graminicola* and the necrotroph *Cochliobolus heterostrophus* [70, 71].

Two proteins from *C*. *miyabeanus*, CM_2749 and CM_6024, have similarity to the Nep1-like (necrosis and ethylene inducing peptide-like or NLPs) family of proteins. In some fungal pathogens NLPs are expressed *in planta* between the biotrophic and necrotrophic phase in dicot hosts [72, 73], or at the end of the symptomless phase in some monocot hosts [74]. NPLs elicit a hypersensitive-like response in dicots [75]. In monocots, however, NLPs do not appear to induce similar responses as the disruption of *NLP* does not affect pathogenicity or virulence; instead, a role in microbe inhibition has been suggested. CM_6024 homologs are present in *C*. *sativus*, *C*. *heterostrophus* and also in *Collectrichum higginsianum*, where a necrosis-inducing protein (ChNLP1) is expressed only during the switch to the necrotrophic phase in *Nicotiana benthamiana* [71].

Other larger secreted proteins with potential roles in pathogenicity included predicted cutinases, peptidases, fungal transporters belonging to the major facilitator superfamily (MFS), and ATP-binding cassette (ABC) group proteins, in addition of CAZymes. Some of these are described in further detail below.

### Carbohydrate-active enzymes (CAZymes)

Fungal plant pathogens need access to nutrients located mostly in host cell protoplasts. Thus, they secrete a wide array of proteins to breach plant barriers (i.e. cuticle and cell walls) and degrade complex carbohydrates for carbon acquisition. The *Cm*TG12bL2 genome encodes 604 carbohydrate binding- and catalytic protein-modules distributed in 530 protein-coding genes. They are involved in binding, modification and breakdown of plant carbohydrates, fungal cell wall biosynthesis, remodeling and turn over, as well as *N*- and *O*-glycoprotein synthesis and processing. The number of CAZymes identified was in the upper range of those found in other Ascomycetes (Table 3) and substantially higher than in *Saccharomyces cerevisiae*, the obligate biotroph *Blumeria graminicola*, the hemibiotroph *Mycosphaerella graminicola*, and the necrotrophs *Pyrenophora tritici-repentis*, *P*. *teres* f. *teres*, *Leptosphaeria maculans*, and *Alternaria brassicicola*, but close to the number found in the hemibiotrophs *Gibberella zeae* and *Magnaporthe oryzae*, and the necrotrophs *Phaeosphaeria nodorum* SN15 and *Cochliobolus heterostrophus*. The number and variety of *Cm*TG12bL2 CAZymes are within the range of those found in other *Cochliobolus* species without considering CAZyme auxiliary activity modules [28].

**Table 3:** Comparison of predicted carbohydrate protein modules present in 12 Ascomycete genomes.

Species^a^	Glycoside hydrolases	Glycosyl-transferases	Polysaccharide lyases	Carbohydrate esterases	Auxiliary activities	Carbohydrate-binding modules	Total^b^
*S*. *cerevisiae*	52	67	0	2	7	15	143
*B*. *graminicola*	66	56	0	9	7	14	152
*M*. *graminicola*	195	105	3	16	47	27	393
*P*. *tritici-repentis*	233	100	10	39	92	52	526
*L*. *maculans*	222	99	19	36	83	64	523
*A*. *brassicicola*	243	93	24	43	83	63	549
*P*. *teres* f. *teres*	236	99	10	39	69	61	514
*P*. *nodorum*	253	90	10	49	100	61	563
*G*. *zeae*	245	107	20	45	72	89	578
*C*. *miyabeanus*	253	101	14	50	99	87	604
*M*. *oryzae*	259	102	5	51	93	119	629
*C*. *heterostrophus*	274	104	15	49	89	102	633

^a^*S*. *cerevisiae*: S*accharomyces cerevisiae* S288c, *B*. *graminicola*: *Blumeria graminicola* f. sp. *hordei*, *M*. *graminicola*: *Mycosphaerella graminicola*, *P*. *tritici-repentis*: *Pyrenophora tritici-repentis*, *L*. *maculans*: *Leptosphaeria maculans*, *A*. *brassicicola*: *Alternaria brassicicola*, *P*. *teres* f. *teres*: *Pyrenophora teres* f. *teres*, *P*. *nodorum*: *Phaeosphaeria nodorum* SN15, *G*. *zeae*: *Gibberella zeae*, *C*. *miyabeanus*: *Cochliobolus miyabeanus* TG12Lb2, *M*. *oryzae*: *Magnaporthe oryzae* 70–15, *C*. *heterostrophus*: *Cochliobolus heterostrophus* C5.

^b^Total is the sum of all CAZYmes (binding and catalytic protein-modules).

We identified 69, 29, three, and nine families of glycoside hydrolases (GH), glycosyltransferases (GT), polysaccharide lyases (PL), and carbohydrate esterases (CE), respectively, in addition to 12 families of auxiliary activity (AA) enzymes [76] in the *Cm*TG12bL2 genome. There are 13 families of non-catalytic carbohydrate binding modules (CBMs) (S5 Table) in addition to five proteins with distant similarity to plant expansins. More than 30 *Cm*TG12bL2 genes had combined catalytic and non-catalytic CAZy modules.

A wealth of auxiliary activities (AA) enzymes (99) was identified with diverse catalytic functions that facilitate plant lignocellulose degradation. The most prominent families were AA1, AA3, and AA9. All three subfamilies of AA1 were found containing laccases, ferroxidases, and laccase-like multicopper oxidases (LMCOs), respectively. Additional details of *Cm*TG12bL2 CAZymes are provided in S1 Text [77, 78, 79, 80, 81, 82, 83, 84].

**Fig 1:**
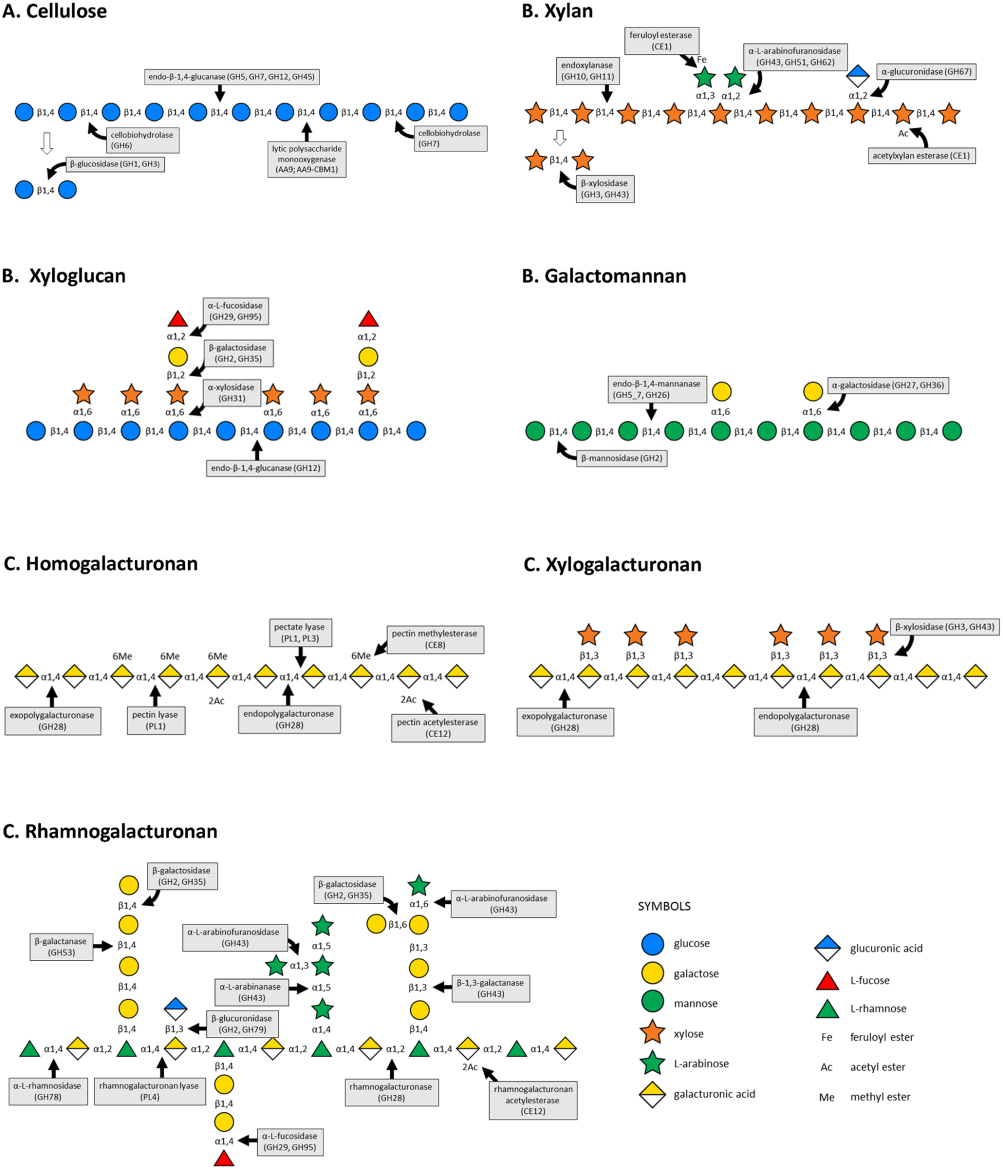
Schematic representation of hypothetical degradation of wildrice cell wall polysaccharides by CAZymes. A. Degradation of cellulose by cellulolytic enzymes: endo-β-1,4-glucanase, cellobiohydrolase, β-glucosidase, and lytic polysaccharide monooxygenase; B. Degradation of hemicellulose polysaccharides and substituted residues by hemicellulolytic enzymes. *Xylan*: endoxylanase, β-xylosidase, acetylxylan esterase, feruloyl esterase, α-L-arabinofuranosidase, α-glucuronidase; *Xyloglucan*: endo-β-1,4-endoglucanase, α-L-fucosidase, β-galactosidase, α-xylosidase; *Galactomannan*: endo-β-1,4-mannanase, β-mannoside, α-galactosidase. C. Degradation of pectin polysaccharides and substituted moieties by pectinolytic enzymes. *Homogalacturonan*: endo- and exopolygalacturonase, pectin and pectate lyase, pectin acetylesterase, pectin methylesterase; *Xylogalacturonan*: endo- and exogalacturonase, β-xylosidase; *Rhamnogalacturonan*: rhamnogalacturonase, rhamnogalacturonan lyase, rhamnogalacturonan acetylesterase, α-L-rhamnosidase, β-1,4-galactosidase, β-1,4-galactanase, β-glucuronidase, α-L-arabinofuranosidase, α-L-arabinanase, β-1,6-galactosidase, β-1,3-galactanase α-L-fucosidase. The glycan symbol nomenclature used was according to [85].

To infect and obtain nutrients from wildrice tissues, *C*. *miyabeanus* needs to breach the cuticle and cell walls composed of cellulose, cross-linking glycans (hemicellulose), and pectin. Numerous CAZymes capable of degrading the backbone chains of those layers as well as removal of substitution residues for complete and efficient degradation, were predicted through electronic annotation (blastp, 1E-22) (S6 Table). A schematic representation of the possible function of some of the *Cm*TG12bL2 CAZymes that potentially allows the fungus to breach the structural barriers of wildrice tissues and acquire nutrients to sustain plant colonization is presented in Fig 1 [85].

### Nonribosomal peptide synthetases and polyketide synthases

Fungi produce a plethora of natural products that include NRPs, polyketides, terpenoids, and alkaloids, among others. They play important roles in growth and development, reproduction, response to oxidative stress, and pathogenicity toward plants and microorganisms [86]. NRPs are synthesized through a ribosome-independent pathway by mono- or multimodular NRPSs where each module is composed of a set of core domains. The minimal module consists of an adenylation (AMP) domain, a peptidyl (P) carrier protein or thiolation (T) domain and a condensation (C) domain whose biochemical functions are described elsewhere [86]. In some enzymes the C domain is replaced by a thioesterase NADP(H) dependant reductase domain [40]. Additional domains for epimerization and methylation may be present in the enzyme, adding diversity to the final polypeptide chain [87]. Previous phylogenetic analysis indicated that mono- and bimodular subfamilies contain more conserved domain structures and may have originated earlier than enzymes with more modular composition [40].

In the *Cm*TG12bL2 genome we identified one α-aminoadipate reductase (AAR) and twelve putative NRPSs (Table 4), including proteins with homology to Ch_NPS2, Ch_NPS4, Ch_NPS6, and Ch_NPS10. The last four are the most conserved among Dothidiomycetes and are involved in virulence, morphology, cell surface hydrophobicity, sensitivity to stresses, and fitness [88]. CM_4358 has a monomodular structure similar to Ch_NPS10, with A, T, and NAD binding domains and a short-chain dehydrogenase, which confers substrate specificity and provides a catalytic site. Mutations in the latter gene affect colony morphology and tolerance to oxidative stress [86]. *Ch_NPS10* has the highest rate of conservation among 18 Dothidiomycetes [28]. CM_8122 is a multimodular enzyme with a similar structural organization to Ch_NPS2. The latter catalyzes the synthesis of an intracellular siderophore (ferricrocin) involved in iron storage and essential for development of asci and ascospores in *C*. *heterostrophus* [89] but not required for virulence in maize. CM_10529 is highly similar to Ch_NPS4, which is also well conserved among filamentous ascomycetes and is involved in cell surface hydrophobicity in *C*. *heterostrophus*, *Alternaria brassicicola*, and *Giberella zeae*, and also plays a role in conidial cell wall development and rate of germination in *A*. *brassicicola* [90]. CM_1661 has high similarity to Ch_NPS6 (Table 4), an extracellular siderophore that in *C*. *heterostrophus*, as well as its homologs in a rice-infecting strain of *C*. *miyabeanus*, and other filamentous ascomycetes are involved in virulence. In *C*. *heterostrophus*, NPS6 also confers tolerance to oxidative stress [42], and it is thought to supply iron to the fungus during host colonization [55]. CM_194 and CM_5231 are similar to Ch_NPS12, but the latter *Cm*TG12bL2 protein has a lower percent identity and lacks the ferric reductase-like membrane component (Pfam01794) and instead has a spore coat protein U domain (pfam05229) usually found in bacterial NRPSs. CM_10197 has homology to the hybrid NRPS/PKS protein Ch_NPS7/PKS24 (Table 5). CM_10647 is like an AAR, proteins that are phylogenetically close to multimodular NRPSs and involved in lysine biosynthesis in fungi [40]. CM_8006 has similarity to a less conserved NRPS in *Cochliobolus* species, the multimodular Ch_NPS3. Three additional NRPS-like proteins (CM_9450, CM_4038, and CM_6570) were found that are similar to hypothetical proteins of *C*. *heterostrophus* C5 and *C*. *miyabeanus* WK-1C.

**Table 4:** Identification and structure of nonribosomal peptide synthetases in *Cochliobolus miyabeanus* TG12bL2.

CM ID number^a^ (amino acids)	NRPS homolog (organism; accession number)	Percent protein coverage^b^	*e*-value^b^	Percent identity^b^	Predicted modular organization of *C*. *miyabeanus* proteins^c^
CM_8122 (5394)	NPS2 (Ch; AAX09984)	99	0	86	A[T]C-ATC-ATC-ATC TCTC
CM_10529 (7224)	NPS4 (Ch; AAX09986)	100	0	93	TC-C-ATC-ATC-C-ATC-ATC-CT-CT
CM_1661 (1939)	NPS6 (Ch; AAX09988)	90	0	94	ATC-(dA)TTC
CM_4358 (1282)	NPS10 (Ch; AAX09992)	100	0	97	ATN D
CM_8006 (5142)	NPS3 (Ch; AAX09985)	100	0	92	ATC-AMTC-ATC-A[M]TC
CM_10197 (2542)	NPS7 (Ch; AAX09989)	99	0	88	^d^
CM_194 (1006)	NPS12 (Ch; AAX09994)	100	0	94	A [R]
CM_5231 (1017)	NPS12 (Ch; AAX09994)	100	0	55	A [SC]
CM_3163 (3908)	NPS1/NPS3/NPS13 expanded NPS-like	-	-	-	C-ATC-A[M]TC-ATC-d(A)
CM_10647 (1179)	AAR-like (Cm; XP007682966)	100	0	100	ATN
CM_9450 (1051)	NPS-like (Ch; EMD86802)	98	0	89	A[T]N
CM_4038 (1218)	NPS-like (Cm; XP007686974)	100	0	98	ATN-AAA
CM_6570 (1063)	NPS-like (Cm; XP007692684)	100	0	100	ATN

^a^ *Cochliobolus miyabeanus* TG12bL2 protein identification number.

^b^ Percent of protein coverage in pairwise alignment, *e*-value and percent of identity determined using BLAST.

^c^ Predicted domain structural organization based on similarity to the HMMER database (http://hmmer.janelia.org/) with a cut-off value = 0.01 and hit = 0.03.

Domains within brackets were detected with cut off > 0.01 and ≤ 1, parentheses indicate domains detected after manual inspection based on conserved amino acids. A = AMP-binding (pfam00501); T = PP (pfam00550), phosphopantetheine attachment site or thiolation domain (or peptidyl carrier protein); C = condensation domain (pfam00668), N = NAD_binding_4 (pfam07993), D = short chain dehydrogenase (pfam00106), M = methyltransferase domain (pfam13847), R = Ferric reductase-like membrane component (pfam01794), SC = spore coat protein U domain (pfam05229), dA = putative degenerated A2 domain, AAA = ATPases associated with diverse cellular activities (pfam13671). AAR = α-aminoadipate reductase. Ch = *C*. *heterostrophus*; Cm = *C*. *miyabeanus* ATCC 44560.

^d^ Structural domain organization of NPS7/PKS24 hybrid protein CM_10197 described in Table 5.

**Table 5:** Identification and structure of polyketide synthases in *Cochliobolus miyabeanus Cm*TG12bL2.

CM ID number^a^ (amino acids)	PKS homolog (organism; accession number)	Percent protein coverage^b^	*e*-value^b^	Percent identity^b^	Predicted modular organization of *C*. *miyabeanus* proteins^c^
CM_4294 (2487)	PKS3 (Ch; AAR90258)	100	0	96	KS-AT-DH-ME-ER-KR-PP
CM_1415 (2639)	PKS5 (Ch; AAR90260)	100	0	94	KS-AT-DH-ME-ER-KR-PP
CM_1147 (2550)	PKS6 (Ch; AAR90261)	95	0	93	KS-AT-DH-ME-ER-KR-PP
CM_2466 (2335)	PKS7 (Ch; AAR90262)	99	0	32	KS-AT-DH-ER-KR-PP
CM_416 (2621)	PKS8 (Ch; AAR90263)	97	0	96	KS-AT-DH-ME-ER-KR-PP
CM_9255 (2274)	PKS9 (Ch; AAR90264)	100	0	91	KS-AT-DH-ER-KR-PP
CM_6592 (2468)	PKS12 (Ch; AAR90267)	100	0	84	KS-AT-DH-ME-ER-KR-PP
CM_466 (2155)	PKS14 (Ch; AAR90268	98	0	91	KS-AT-DH-ME-ER-KR-PP
CM_10206 (2441)	PKS15 (Ch; AAR90269)	93	0	91	KS-AT-DH-ME-ER-KR-PP
CM_5798 (2155)^d^	PKS18 (Ch; AAR90272)	99	0	96	KS-AT-DH-PP-PP-Th
	PKS1 (Cm, BAD22832)	100	0	99	
CM_8343 (1784)	PKS19 (Ch; AAR90273)	100	0	94	KS-AT-DH-PP
CM_4293 (2583)	PKS21 (Ch; AAR90275)	84	0	97	KS-AT-PP-ME-N
CM_6273 (2715)	PKS22 (Ch; AAR90276)	86	0	88	KS-AT-DH-PP-PP-PP-ME-abH-Pep
CM_10241 (2232)	PKS23 (Ch; AAR90277)	92	0	85	KS-AT-DH-PP-PP-ME
CM_10197 (2542)	PKS24 (Ch; AAR90278)	99	0	89	AMP-PP-KS-AT-KR-PP-N
CM_11065 (2249)	PKS14 (Cs, EMD68395)	100	0	89	KS-AT-DH-PP-PP-N
CM_1155 (2465)	PKS6 (Cs; EMD66380)	100	0	93	KS-AT-DH-ME-ER-KR-PP
CM_1154 (1987)	PKS16 (Cs, EMD66379)	88	0	100	KS-AT-DH-PP-TE-UbiA
CM_803 (1802)	PKS13 (Cs; EMD66882)	100	0	92	KS-AT-PP

^a^ *Cochliobolus miyabeanus* protein identification number.

^b^ Percent of protein coverage in the pairwise alignment.

^c^ Predicted domain structural organization based on similarity to the CDD (http://www.ncbi.nlm.nih.gov/Structure/cdd/cdd.shtml) with a cut-off *e*-value = 0.01, and/or HMMER database (http://hmmer.janelia.org/) with a gathering cut-off value Model = 0.01 and hit = 0.03.

^d^ PKS18 (AAR90272) and PKS1 (BAD22832) from *C*. *heterostrophus* and *C*. *miyabeanus*, respectively are both homologs proteins of CM_5798.

KS = beta-ketoacyl-synthase domain (pfam00109); AT = acyl transferase domain (pfam00698); PP = phosphopantetheine attachment site (pfam00550 or smart00823); DH = polyketide synthase dehydratase domain (pfam14765); ME = methyltransferase domain (pfam08242); ER = enoyl reductase domain (cd05195) or ADH_N, alcohol dehydrogenase (pfam08240) and ADH_Zinc_N, zinc-binding dehydrogenase (pfam00107), TE = thioesterase (pfam00975), N = NAD_binding_4 (pfam07993), abH = alpha/beta hydrolase (pfam07859), Pep = Peptidase (pfam00326); A = AMP-binding (pfam00501). PKS = polyketide synthase, Hp = hypothetical protein. Ch = *Cochliobolus heterostrophus*, Cs = *Cochliobolus sativus*, Cm *= Cochliobolus miyabeanus*.

A phylogenetic tree was constructed to analyze the conservation and relationships among AMP domains present in the *Cm*TG12bL2 NRPS proteins with those of other *Cochliobolus* species (S5 Fig). Within the NRPS conserved group, the single AMP domain of each *Cm*TG12bL2 NPS6, NPS7/PKS24, NPS10, the two NPS12, and the related adenylating enzyme AAR, clustered with their *Cochliobolus* counterpart with well supported bootstrap values (S5 Fig). Each of the four AMP domains of *Cm*TG12bL2 for NPS2 and NPS4 proteins grouped with the corresponding domain in the other three *Cochliobolus* isolates. This was also the case for the AMP domains of the *Cm*TG12bL2 homolog of less conserved multimodular NPS3. The monomodular NPS11 was not present in *Cm*TG12bL2, nor was it found in *C*. *miyabeanus* WK-1C [24]. However, the single NPS11 AMP domain of both *C*. *heterostrophus* strains grouped with the first AMP domain (AMP1_2) of the two bimodular NRPSs: GliP, responsible for gliotoxin production in *A*. *fumigatus* (EAL88817), and SirP, responsible for sirodesmin biosynthesis in *Leptosphaeria maculans* (AAS92545). Interestingly, both strains of *C*. *miyabeanus* each contain a NRPS-like protein, CM_2059 in *Cm*TG12bL2 and ID 107726 in *C*. *miyabeanus* WK-1C, with a single AMP domain that groups instead with the second module (AMP2_2) of both GliP and SirP proteins (S5 Fig).

In *Cochliobolus* species, NPS1, NPS3, and NPS13 proteins are discontinuously distributed. Their AMP domains display duplications/deletions, fusions, and/or recombinations that are thought to give rise to novel NRPSs within these expanded clades [24]. CM_3163 is a tetra-modular protein that has a homolog in *C*. *miyabeanus* WK-1C (W6YWH5) and belongs to this expanded group. We analyzed the placement of the AMP domains with regard to those in *C*. *miyabeanus* WK-1C, and the two *C*. *heterostrophus* strains (C4 and C5) (S5 Fig). Each AMP domain of the NPS1/NPS3/NPS13 NRPS-like proteins from both *C*. *miyabeanus* isolates always clustered together. Additionally, the AMP1_4 of CM_3163 grouped with maximum bootstrap support (100%) to AMP1_1 of the pseudogene *Ch_NPS13* while AMP2_4 and AMP3_4 of CM_3163 clustered with AMP2_4 of Ch_NPS3. The fourth AMP domain of CM_3163 was weakly related to AMP3_4 of Ch_NPS3 (S5 Fig). Homologous AMP domains from Ch_NPS1 are absent in both *C*. *miyabeanus* isolates. The AMP1_1 domains of NPS-like proteins (CM_9450, CM_4038, CM_6570) were located with the outgroup set, and grouped within a strongly supported subset of AMP domains of *Fusarium graminearum* and *Aspergillus* species involved in ochratoxin biosynthesis (S5 Fig).

Most fungal polyketides are synthesized by type I PKSs and are monomodular enzymes with multiple catalytic domains carrying out repeated biosynthetic reactions. These rapidly evolving proteins catalyze the condensation of Co-enzyme A, either from CoA thioesterified carboxylic acids in reducing PKSs, or from acetyl- or malonyl-CoA in nonreducing PKSs [41] to form carbon chains or cyclic forms of varying lengths. A minimal module of PKS consists of a β-ketosynthase (KS), acyltransferase (AT), and acyl carrier protein (ACT) or PPT attachment site (PP) whose roles in the enzyme have been previously described [41]. Additional modules with specific functions, β-ketoreductase (KR), dehydrogenase (DH), enoyl reductase (ER), methyltransferase (ME), and thioesterase (TE) add modifications or cyclize the polyketide compounds. Based on the modules present, the PKSs can be subdivided into reducing or non-reducing enzymes, the latter lacking some or all of the reducing domains (KR, DH, and ER) [41].

Nineteen PKSs were identified in the *Cm*TG12bL2 genome. Overall, 15 proteins matched the *C*. *heterostrophus* C4 PKS set (Ch_PKS). Those included ten (Ch_PKS3, Ch_PKS5, Ch_PKS9, Ch_PKS12, Ch_PKS14, Ch_PKS15, Ch_PKS18, Ch_PKS19, Ch_PKS23, and Ch_PKS24) that are common to all *Cochliobolus* species and additional five (Ch_PKS6, Ch_PKS7, Ch_PKS8, Ch_PKS21, and Ch_PKS22) with a discontinuous distribution among the species of the genus. Four others are homologs of PKS6, PKS13, PKS14, and PKS16 of *C*. *sativus* (Table 5). Predictions of protein domains, excluding PKS24, indicate that eight PKSs have the seven ancestral modules of the fully reduced type I PKSs, nine are partially reducing (lacking one or more DH, KR, or ER domains), and one is a non-reducing type. In the *Cm*TG12bL2 genome, two of the 21 PKSs reported for *C*. *miyabeanus* WK-1C were not detected, including the duplicated and expanded PKS14. Overall, the number of *PKSs* and *NRPSs* found in the *Cm*TG12bL2 genome was similar to the number in other members of the Pezizomycotina, and the organization of structural domains are identical or similar to those found in other *Cochliobolus* species.

### Predicted transporter and detoxification systems

Cytochrome P450 monooxygenases (CYPs) are heme-thiolate enzymes involved in degradation and detoxification of xenobiotics [91]. CYPs catalyze chemical modifications in lipophilic compounds from primary and secondary metabolism to create more hydrophilic derivatives. We identified 113 CYPs in the *Cm*TG12bL2 genome (S7 Table), similar to the number found in other members of the Pezizomycotina [92]. Examples of enzymes predicted to participate in primary metabolism include eburicol 14-α demethylase (CM_837) that is a member of the CYP51 family, a sterol C-22 saturase from the fungal-specific CYP61 family (CM_7374) involved in membrane ergosterol biosynthesis, members of the CYP52 family (CM_10978, CM_255, and CM_1621) that catalyze initial degradation of n-alkanes and fatty acids, and a CYP56 family member (CM_6988) that participates in formation of the N, N’-bisformyl dityrosine spore cell wall component [91]. The *Cm*TG12bL2 genome encodes CYPs from families known to be involved in biosynthesis of aflatoxins (CYP59 and CYP62), fumonisins (CYP65 and CYP505), tricothecenes (CYP65, CYP68, and CYP526), and gibberellin (CYP68) (S7 Table). Additionally, other CYPs could be involved in detoxification and degradation of xenobiotics. For example, CM_10633 is similar to one of the pisatin demethylase genes, PDA6-1 from *Nectria hematococca* (CYP57) involved in degrading the pterocarpan phytoalexin produced by *Pisum sativum* [93]. CM_8068 is a likely ortholog of CYP53A15 from *C*. *lunatu*s, a benzoate para-hydroxylase with O-demethylase activity that catalyzes degradation of benzoic acid and derivatives as well as natural toxins [94]. CM_6446, a CYP504A member, and CM_5407 from family CYP504B have similarity to phacA, a phenylacetate 2-hydrolase from *Aspergillus nidulans* [95] and to phacB, a 3-hydroxyphenylacetate 6-hydrolase [96], respectively. Both participate in phenylacetate degradation to Krebs cycle intermediate compounds.

**Fig 2:**
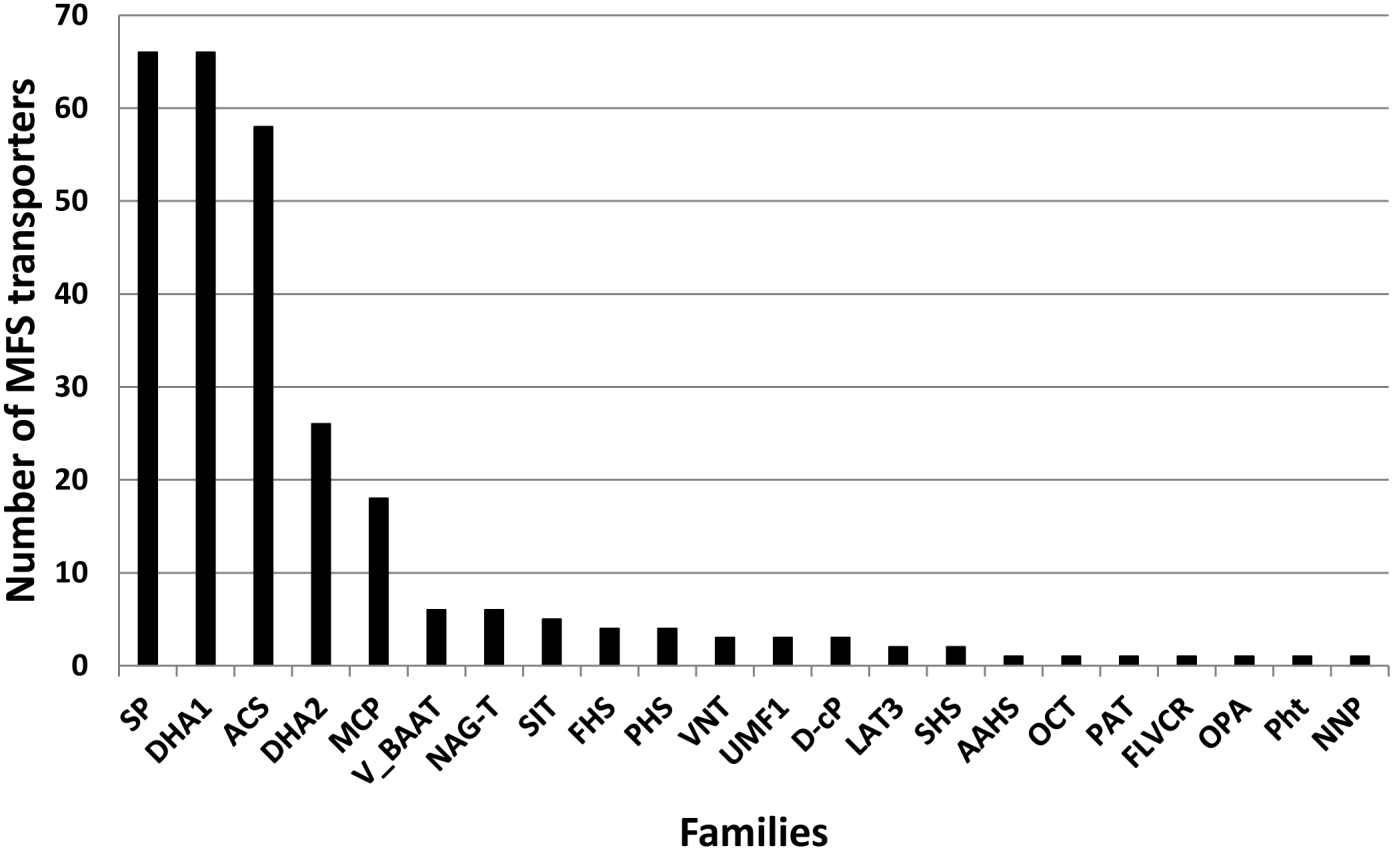
Predicted families within the major facilitator superfamily transporters in the *Cm*TG12bL2 genome. SP: Sugar Porter (2.A.1.1), DHA1: Drug:H^+^ Antiporter-1 (12 Spanner) (2.A.1.2), ACS: Anion:Cation Symporter (2.A.1.14), DHA2: Drug:H^+^ Antiporter-2 (14 Spanner) (2.A.1.3), MCP: Monocarboxylate Porter (2.A.1.13), V-BAAT: Vacuolar Basic Amino Acid Transporter (2.A.1.48), NAG-T: N-Acetylglucosamine Transporter (2.A.1.58), SIT: Siderophore-Iron Transporter (2.A.1.16), FHS: Fucose: H^+^ Symporter(2.A.1.7), PHS: Phosphate: H^+^ Symporter (2.A.1.9), VNT: Vesicular Neurotransmitter Transporter (2.A.1.22), UMF1: Unknown Major Facilitator-1 (2.A.1.24), D-cP: Domain-containing Protein (2.A.1.40), LAT3: L-Amino Acid Transporter-3 (2.A.1.44), SHS: Sialate:H^+^ Symporter (2.A.1.12), AAHS: Aromatic Acid:H^+^ Symporter (2.A.1.15), OCT: Organic Cation Transporter (2.A.1.19), PAT: Peptide-Acetyl-Coenzyme A (2.A.1.25), FLVR: Feline Leukemia Virus Subgroup C Receptor (2.A.1.28), OPA: Organophosphate:P_i_ Antiporter (2.A.1.4), Pht: Proteobacterial Intraphagosomal Amino Acid (2.A.1.53), NNP: Nitrate/Nitrite Porter (2.A.1.8).

Fungal transporters provide resistance to a variety of drugs, exogenous mycotoxins and fungicides, and contribute to pathogenicity by delivering mycotoxins outside fungal cells [97]. In the *Cm*TG12bL2 genome, as in other Ascomycetes, the major facilitator superfamily (MFS) and the ATP-binding cassette (ABC) superfamily, with 279 and 46 genes respectively, account for most of the transporters identified. The *Cm*TG12bL2 MFS transporters are distributed in 22 families (Fig 2). Five families have the majority of the predicted MFS proteins: the sugar porter (SP), the Drug:H^+^ antiporter-1 (12 Spanner) (DHA1), the drug:H^+^ antiporter-2 (14 Spanner) (DHA2), the monocarboxylate porter (MCP) and the anion:cation symporter (ACS). Examples of MFS found include a monosaccharide transporter similar to MstC, the low affinity glucose:H^+^ symporter from *A*. *niger* [98], and a high-affinity xylose–proton symporter GXS1 [99], a protein with similarity to Flr1 of *Saccharomyces cerevisiae*, a MFS involved in resistance to the antifungal drug fluconazole and several chemically unrelated drugs [100], a nicotin acid permease, and H^+^:biotin symporter involved in uptake of vitamins [101].

**Fig 3:**
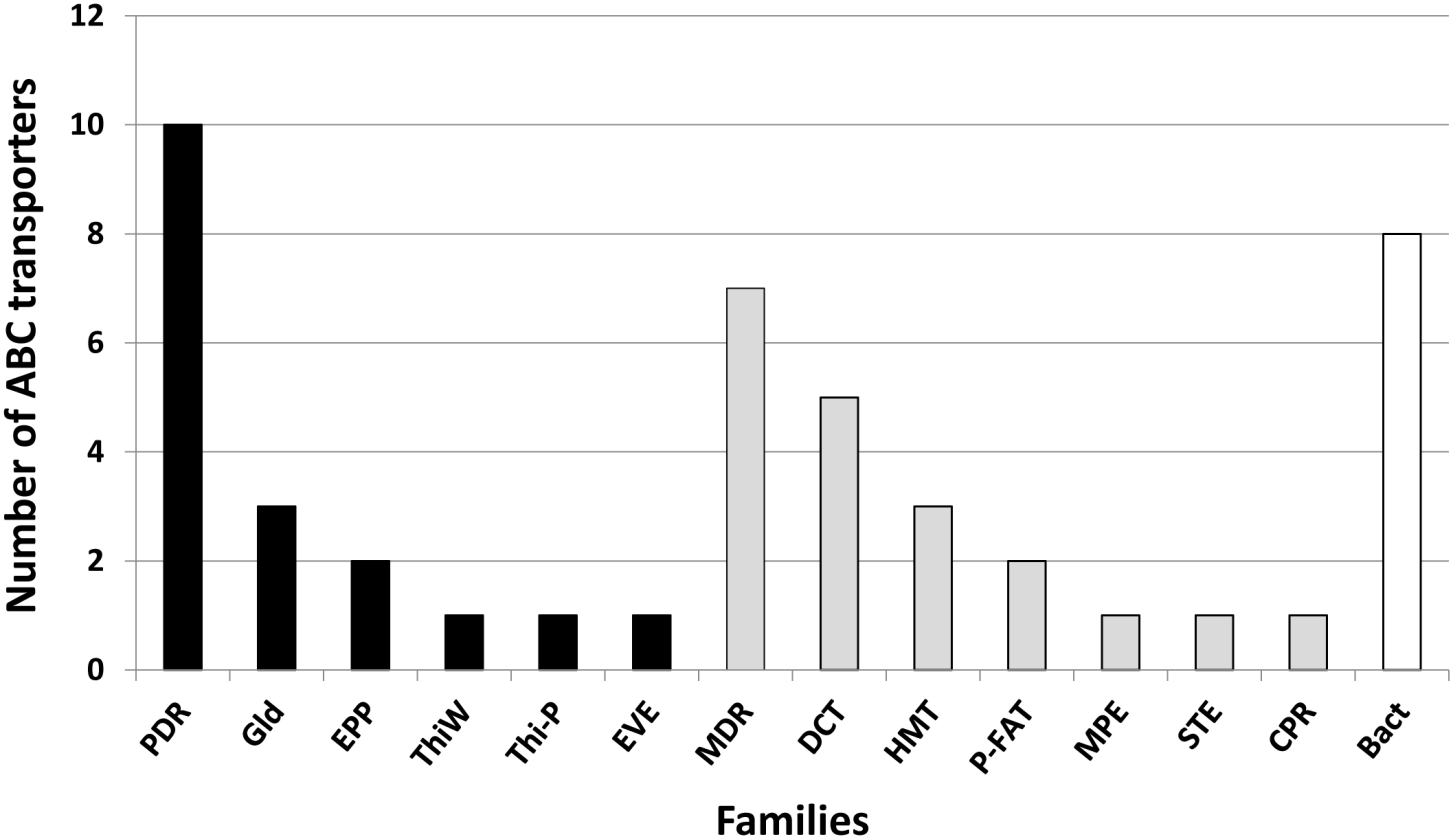
Predicted families of ABC transporters in the *Cm*TG12bL2 genome. Black bars: ABC2 superfamily that includes: PDR, Pleiotropic Drug Resistance; Gld, Gliding Motility ABC transporter; EPP, Eye Pigment Precursor transporter; ThiW, Putative Thiamine Uptake transporter; ThiP, Thiamine Precursor; EVE, Ethyl Viologen Exporter. Gray bars: ABC1 superfamily with MDR, MultiDrug Resistance exporter; DCT, Drug Conjugate Transporter; HMT, Heavy Metal Transport; P-FAT, Peroxysomal Fatty Acyl-CoA Transporter; MPE, Mitochondrial Peptide Exporter; STE, α-Factor Sex Pheromone Exporter; CPR, Cholesterol/Phospholipid/Retinal (CPR) Flippase Family. White bar: Bact, Bacterial-like transporters.

Transporters within the ABC superfamily typically have two homologous components each containing a nucleotide-binding domain (NBD) and six transmembrane-spanning helices that form the transmembrane domain (TMD_6_); however, half-size transporters are also found [97, 102]. Most of the *Cm*TG12bL2 ABC transporters are in the ABC1 and ABC2 superfamilies (Fig 3), with the majority within the families that participate in efflux of toxins [103]. Predicted *Cm*TG12Lb2 proteins are similar to those in the multidrug resistance exporter family, ABCB (i.e., Mdr1 from *A*. *fumigatus* that confers resistance to antifungal compounds [97]), and the pleiotropic drug resistance family ABCG (i.e., AtrB transporter of *A*. *nidulans* associated with resistance to cycloheximide, a variety of fungicides, and toxins including antifungal plant compounds [102]). Additional *Cm*TG12Lb2 proteins within the ABCG family have similarity to CDR1 and CDR2 of *Candida albicans*, that play a role in resistance to antifungal azoles; and to STS1, the suppressor of sporidesmin toxicity, of *Saccharomyces cerevisiae* that confers resistance to sporidesmin and others drugs such as cycloheximide [104]. Putative homologs of the metal resistance protein YCF1 from *S*. *cerevisiae* in the ABCC family are also present.

### *C*. *miyabeanus* transcriptome *in planta*

As expected, the vast majority of the reads generated were of plant origin. The totality of reads mapped to the *Cm*TG12bL2 genome yielded, depending on the particular replicate, 1.6% to 3.0% fungal reads. Pairwise correlation coefficient for log_2_ transformed RPKM between biological replicates ranged from r = 0.87 to r = 0.93, indicating low variation between samples. The average number of *Cm*TG12bL2 transcripts at 48 hai across replications was 10,787 ± 70, of which 10,674 that were present in at least three out of the four replicates, were selected for further analyses. Average log_2_ transformations of the transcripts showed a double exponential distribution (data not shown). For the enrichment analysis (Fisher test), we only investigated transcripts (1,039) located in the three upper quartiles of the distribution.

### Functional categorization of fungal transcripts

Over 5,700 gene ontology (GO) terms were assigned to the 10,674 transcripts. Annotations were found for 17 categories within the *biological process* domain and the most populated were metabolic processes, cellular processes, single-organism processes and localization. The highest number of transcripts for the *molecular function* domain was in binding and catalytic activities; and within the *cellular component* domain was in cell, organelle, and membrane functions (S6 Fig).

### Gene expression and enrichment analysis

We further investigated the highest expressed genes in the upper 10% quartile. Those with predicted functions are involved in protein synthesis (40S and 60S ribosomal proteins) and protein modification (heat shock proteins), translation (eukaryotic translation initiation factor 2), signaling (14-3-3 proteins, calmodulin, MAPK), and transport of secretory proteins (SEC14 cytosolic factor). Transcripts present in high numbers or in the enriched or over-represented pool are associated with reactive oxygen species scavenging, such as superoxide dismutases (t_Cm_4737, t_Cm_8015), glutathione peroxidase (t_ Cm_5661), peroxiredoxins (t_Cm_5333, t_Cm_1899), and catalases (t_ Cm_1931, t_Cm_4481, t_Cm_5915). Transcripts involved in signaling through regulation of oxidative stress systems such as mitogen-activated protein kinase HOG1 (t_Cm_6356) and putative homologs of ChAp1 transcription factors (t_Cm_3085) and Skn7 (t_Cm_9152) were also found. Other known stress-related genes highly expressed in *Cm*TG12bL2 included cytochrome c (t_Cm_10384), ubiquitin-conjugating enzyme E2 N (t_Cm_2552), peptidyl-prolyl cis-trans isomerase (t_Cm_4487, t_Cm_6343), ribosomal protein S5 (t_Cm_2988), and protein phosphatase PP2A (t_Cm_2839). A salicylate hydroxylase (t_Cm_9653) was also over-represented.

Seventy-four CAZymes genes were highly expressed, of which 45 were over-represented transcripts. The vast majority were GH (51%) with similarity to cellulase (GH7: t_Cm_1858), mixed-linkage glucanase (GH12: t_Cm_6551), exoglucanases/cellobiohydrolases (GH6: t_Cm_2649; GH7: t_Cm_2863), and putative β-glucosidases (GH3: t_Cm_28, t_Cm_9000). Within the auxiliary group, five AA9 transcripts, with two of them appended to a single CBM1 module were enriched as well. CAZymes that depolymerize other plant cell wall glycans were also over-represented. These included a β-1,4-endoxylanase precursor (GH11: t_Cm_8238), α- and β-galactosidases (GH36: t_Cm_3708; GH35: t_Cm_7042), acetylxylan esterase (CE1: t_Cm_7354), and feruloyl esterase (CE1: t_Cm_6253). Other transcripts encoded enzymes that could degrade pectin such as rhamnogalacturonan acetyl esterase (CE12: t_Cm_4971), pectate lyase (PL3: t_Cm_3311), and pectin esterase (CE8: t_Cm_2172). Additionally, transcripts containing CBM domains like CBM1, CBM35, CBM48 and CBM50 in single or repeated units, present alone and in association with catalytic domains were in the enriched pool. Two putative cutinases (CE5: t_Cm_5545 and t_Cm_6538) were in the enriched set, which could contribute to initiating invasion of wildrice tissues.

We also found 32 SSP-encoding genes with high expression including five that were over-represented and belonged to the GO categories of “extracellular region”, “structural molecular activity”, “heterocyclic compound binding”, and “catalytic activity”. Within the group of effector homologs, only t_Cm_2799 a putative Ecp6 homolog, was over-represented.

No *NRPS* or *PKS* genes were over-represented in the transcriptome at 48 hai, and only PKS7 was within the most expressed genes.

Among CYPs, seven over-represented transcripts were associated with metabolic processes, cell and heterocyclic compound binding. These included monooxygenases from the CYP504 family (t_Cm_6446), putatively involved in phenylacetate degradation, and CYP53 (t_Cm_8068), a benzoate para-hydroxylase, and transcripts of the CYP532 family associated with xenobiotic metabolism, as well as members of families CYP552, CYP68, and CYP52.

Within the overrepresented transporters were 11 predicted MFS members including a glucose/xylose symporter1 (GXS1) (t_Cm_11802), a sugar and polyol (SPT1) and monosaccharide transporters (MstC) (t_Cm_1809 and t_Cm_6329), a STL1 permease (t_Cm_4510), a boron exporter (Atr1) (t_Cm_1340), and nicotinic acid permease (t_Cm_571, t_Cm_8891, t_Cm_9423, t_Cm_7821), among others. Four predicted ABC transporters in the *Cm*TG12bL2 genome were in the enriched pool and had similarity to the multidrug resistance protein CDR1 from *C*. *albicans* (t_Cm_6382) and bacterial-like transporters (t_Cm_ 3444, t_Cm_5374, t_Cm_3853).

### qRT-PCR experiments

Transcript accumulation at 48 hai of the six genes selected from the enriched pool, Ecp6, CYP53, salicylate hydroxylase, β-1,4-endoglucanase, β-1,4-glucosidase, and β-1,4- endoxylanase were validated by qRT-PCR experiments. The relative quantification of the expression of those genes *in planta* at 24 hai and 48 hai was always significantly higher than that *in vitro*. In addition, levels of expression of CYP53, β-1,4- endoxylanase and β-1,4-glucosidase were similar at the two time points considered, while *Ecp6* and salicylate hydrolase were higher at 48 hai compared to 24 hai (S7 Fig), but only the latter was significant. β-1,4-endoglucanase, involved in the degradation of cellulose, was higher at 24 hai. Overall, qRT-PCR expression values were similar to their computed digital counterparts (S7 Fig), thus validating the transcript expressions in the transcriptome analysis at 48 hai.

## Discussion

Here, we report the *de novo* genome assembly of a strain of *C*. *miyabeanus*, *Cm*TG12bL2, isolated from American wildrice. We also assembled and studied the fungal transcriptome during the initial phases of plant infection and colonization. *C*. *miyabeanus* is found infecting *Oryza* species all over the world [5], and in North America it has the potential to cause yield limiting disease on American wildrice [3] and switchgrass [4]. Despite its importance, relatively little is known about the mechanisms of pathogenicity, or host-pathogen interactions [105]. Unlike other *Cochliobolus* pathogens, *C*. *miyabeanus* does not appear to utilize HSTs during plant colonization [24, 106]. In our transcriptome analysis we focused on validating the gene repertoire of the genome analysis and identifying transcripts potentially involved in pathogenicity and others produced in response to host defenses.

The *Cm*TG12bL2 genome is similar to sizes reported for other *Cochliobolus* species. For instance, the draft genome of *C*. *heterostrophus* C4, assembled with Illumina technology was 32.93 Mb, while *C*. *heterostrophus* isolate C5 using Sanger sequencing was 36.46 Mb, and the *C*. *sativus* ND90Pr v.1 genome assembled with a combination of Sanger, 454, and Illumina sequencing yielded 34.42 Mb [24,28]. The length of our assembly is similar to the *C*. *miyabeanus* WK-1C reference genome (31.4 Mb) downloaded from the JGI website (January, 2014; http://genome.jgi.doe.gov/Cocmi1/Cocmi1.download.html) and reported by Condon et al. [106]. Importantly, 92% of the *Cm*TG12bL2 predicted proteins are putative homologous to those in WK-1C, including NPRS, PKS, and most of the SSPs. The majority of the proteins in the set that did not match those in the rice isolate were hypothetical proteins, while the rest (1%) carried known domains and could be associated to DNA replication (DNA polymerases, exonucleases) and repair (Rad51 proteins, endonucleases), retrotransposon activities (retrotransposon gag proteins, integrases, and reverse transcriptase), transcription factors (Zinc-finger, CCHC- and C2H2-types) and proteins of unknown functions (DUF domains). The *Cm*TG12bL2 genome shares a set of proteins with other *Cochliobolus* species that are involved in plant-pathogen interaction, including pathogenicity and virulence. Even though most of them were expressed, and some of them at high levels such as the trypsin-like serine protease Alp1 [61], they were not found statistically over-represented in the transcriptome at 48 hai.

Overall, a relatively large number of the *Cm*TG12bL2 genes (~10%) were annotated as potentially being secreted. Many of those genes have roles in host recognition and/or pathogenicity, and were highly expressed or statistically overrepresented in the transcriptome analysis. Some examples include hydrophobins involved in masking spore recognition by hosts and in sensing the host surface [62], and predicted cutinases that catalyze the disruption of the fatty acid cutin, the main constituent of plant cuticle. The *Cm*TG12bL2 genome harbored a substantial number of gene-encoding CAZymes with more than 8% of their transcripts in the enriched transcriptome pool that could facilitate ingress and colonization of wildrice tissues. Those included enzymes catalyzing reactions for degradation of cellulose, such as endoglucanases, cellobiohydrolases, and β-glucosidases, together with lytic-polysaccharide monooxygenases of family AA9 that are responsible for oxidative cleavage of cellulose. A few AA9 CAZymes are bound to non-catalytic CBM1, credited with providing a more efficient binding to cellulose [83]. Others were enzymes able to depolymerize other plant cell wall glycans, particularly xylan, such as xylanases and β-galactosidases, as well as acetylxylan and feruloyl esterases. The upregulation of some of these genes *in planta* was validated by qRT-PCR analyses. Additionally, a transcript that could be involved in sucrose degradation was highly expressed. Plant sucrose derivatives are used as carbon or energy sources by fungi. Transcripts of predicted MFS transporters putatively involved in sugar uptake and transfer were overrepresented, including a putative homolog of the glucose/xylose symporter1 (GXS1) [99], a low affinity glucose monosaccharide transporter (MstC), [98], and a sugar and polyol transporter (SPT1) that could facilitate growth and adaptation to a variety of nutrient conditions.

A reduced percent of the fungal secretome (17.5%) consisted of small cysteine-rich proteins of less than 200 aa. SSPs are important for pathogenic actions since they can play roles as effectors promoting disease and/or altering host defense mechanisms [107]. Over 96% of all SSPs were proteins that matched those of the rice *C*. *miyabeanus* WK-1C isolate. At 48 hai, 17% of SSPs were highly expressed but only a few were over-represented in the transcriptome enrichment analysis. Within those few having known domains are SSP predicted to be involved in ribosomal activities (guanyl-specific ribonuclease Pb1; translation initiation factor SUI1), assistance in protein folding (peptidyl-prolyl cis-trans isomerase FKBP10), and cell wall stability and resistance to antifungal agents (cell wall mannoprotein PIR3). One particular SSP contained a cerato-platinin (CP) domain. Proteins with CP domains are preferentially located in fungal cell walls, and participate in growth and development in many fungi [108, 109, 110]. Other roles for CP proteins are in plant-fungal interactions not only as elicitors of plant defense mechanisms but also as effectors because they are able to bind chitin and its oligomers, thus, avoiding PAMP-triggered immunity detection during plant invasion [110]. A putative homolog to the extracellular effector Cf_Ecp6 of *Cladosporium fulvum* [67] is overrepresented at 48 hai. The higher expression of this gene *in planta* compared to that *in vitro* was confirmed in our qRT-PCR analysis. Cf_Ecp6 has high affinity for short chitin oligosaccharides and competes efficiently with extracellular plant receptors for degraded chitin fragments preventing activation of PTI in plant hosts [69]. In our transcriptome analysis at 48 hai we identified plant transcripts (data not shown) with very high homology to OsCERK1, the rice chitin elicitor receptor kinase 1. OsCERK1 forms a hetero-oligomer receptor complex with a glycoprotein, chitin-binding receptor CEBiP that upon recognition of chitin fragments triggers production of reactive oxygen species, diterpenoid phytoalexins, and expression of basal defense genes in rice [111, 112]. Even though a transcript similar to CEBiP was not identified at this time point, four other wildrice LysM containing proteins (receptor-like proteins) were expressed. Thus, *C*. *miyabeanus* might avoid elicitation of wildrice defense mechanisms by sequestering chitin fragments.

Proteins synthesized by *NRPS* and *PKS* genes are important in fungal fitness, response to environmental cues, and interaction with other organisms [86]. In other *Cochliobolus* species a great number of these metabolites are HSTs [12,14,21,22,24]. A subset of *NRPS* genes are discontinuously dispersed among *Cochliobolus* species, and could evolve rapidly by recombination, rearrangement and gain/loss of their AMP domains [40] to produce new phytotoxins. For instance, a protein of this class found in *C*. *sativus* pathotype 2, is thought to play a major role in causing virulence on barley cultivar Bowman [24]. One *Cm*TG12bL2 protein (CM_3163) belongs to the rapidly evolving and expandable NPS1/NPS3/NPS13 group and has a homolog in the WK-1C strain. In the phylogenetic analysis, adenylation domains of CM_3163 are closely related to AMP1_1 of Ch_NPS13 (*Cm*TG12bL2 AMP1_4), AMP2_4 of Ch_NPS3 (*Cm*TG12bL2 AMP2_4 and AMP3_4) and AMP3_4 of Ch_NPS3 (*Cm*TG12bL2 AMP4_4). Both, duplication of portions of *NRPS* genes (i.e. *NRPS3*), and either recombination or gene fusion (i.e. *NRPS13*) may have generated the two genes unique to *C*. *miyabeanus* strains. The fact that AMP domains of the mono-modular NPS11 from *C*. *heterostrophus* and a mono-modular NRPS-like gene from *C*. *miyabeanus* clustered separately, the first one with AMP1_2 and the second with AMP2_2 of bi-modular NRPSs GliP of *A*. *fumigatus* and SirP of *Leptosphaeria maculans*, suggests differential retention of AMP domains from an ancestral bi-modular *NRPS* gene.

Overall, 19 PKSs were detected within the *Cm*TG12bL2 predicted set of proteins, including the 10 PKSs common to all *Cochliobolus* species. However, *Cm*TG12bL2 isolate has only one of the two *PKS14* genes found in *C*. *miyabeanus* WK-1C [24]. Overall the number of NRPS and PKS found in our assembly is within the ranges of these proteins reported for *Cochliobolus* species [24]. The wildrice isolate appears to contain the same suite of NPSs and a similar number of PKSs as the rice *C*. *miyabeanus* WK-1C isolate [24] with no additional unique genes.

Cytochrome P450 monooxygenases have been associated with adaptation of fungi to new niches because they catalyze the degradation of chemical substances, and might facilitate pathogenesis [92]. Reduction in phenolic compounds content has been reported in rice leaves at 48 hai with a pathogenic strain of *C*. *miyabeanus* [113]. Three enzymes with homology to benzoate 4-monooxygenases (CYP548, CYP552, and CYP583) and another similar to a benzoate para-hydrolase of family CYP53 were highly expressed during *Cm*TG12bL2 colonization of a wildrice cultivar with improved resistance to FBS. A putative ortholog, *BPH*, from *C*. *heterostrophus* was upregulated during maize infection, suggesting that maize defenses could involve benzoate biosynthesis in [114]. In *C*. *lunatus*, a similar protein (bph) [94], was shown to be a key enzyme in benzoate detoxification in a dose- and time-dependent manner. Thus, some *Cm*TG12bL2 cytochrome P450 monooxygenases could participate in degrading phenolic compounds synthesized by wildrice during fungal infection. A putative salicylate hydroxylase was upregulated at 24 hai and at 48 hai. Similar proteins in *Fusarium graminearum* degrade the signaling molecule salicylic acid, necessary for plant defense [115]. Salicylic acid-induced transcripts were found in the wildrice transcriptome (data not shown) and could be related to defense against the fungus. Identification of fungal genes implicated in the detoxification or inhibition of host compounds is important for identifying the plant defense mechanisms used for counterattack of fungal colonization.

Our results are in agreement with a study of proteomics of *C*. *miyabeanus* during infection on rice [105] and the *Bipolaris sorghicola* transcriptome during sorghum infection [116]. Particularly the findings of fungal oxidative stress activity, expression of CAZymes such as α-L-arabinofuranosidase, xylanase and glucanase, acetylxylan esterase, and LysM domain containing proteins, as well as expression of cutinases, and Alp1 transcripts, suggesting commonalities of virulence mechanisms among *Cochliobolus*/*Bipolaris* species infecting monocots. The *Cm*TG12bL2 genome and transcriptome assemblies and analyses contribute to a better understanding of fungal pathogenicity and open new avenues for targeted mutagenesis in this pathosystem (i.e. cysteine-rich SSP, putative effectors molecules, and CYPs). Further, it offers genomic resources for the interpretation of the *C*. *miyabeanus* infected wildrice transcriptome.

## Conclusions

The genome of *Cochliobolus miyabeanus* isolate *Cm*TG12bL2 pathogenic on wildrice was sequenced using Illumina short-read technology, together with the transcriptome *in planta* after infection. Proteins involved in ROS scavenging, plant tissue degradation and carbohydrate binding, SSPs, a known effector, and detoxification systems were expressed in the infected leaves. This study advances our understanding of fungal pathogenicity on a distant relative of rice, American wildrice. The predicted gene and protein sets will facilitate targeted mutagenesis to infer the functions of pathogenicity and effector genes, as well as comparative transcriptomics of *Cochliobolus* pathogens when colonizing different hosts (i.e. wildrice, switchgrass, and common rice). Further, it may help in refining host breeding strategies for developing more effective genetic resistance against *C*. *miyabeanus*.

## Supporting Information

S1 Fig Length of N_50_, total draft genome, and maximum scaffold with varying *k*-mer values before parameters optimization. A. Length of N_50_ values at *k*-mers varying from 31 to 91 nucleotides increasing by 2 nucleotides. Black column indicates the longest N_50_ length value (75,371bp) for 65 *k*-mer. B. Total length of the draft *C*. *miyabeanus* genome (Mbp) produced by assemblies with increased *k*-mer lengths from 31 to 91 nucleotides. Black column indicates initial genome draft size (31,696,836 bp) for 65 *k*-mer. C. Black column indicates maximum scaffold size (348,044 bp) for *k*-mer 65.
(DOCX)

S2 Fig Distribution of contig-weighted coverage of *Cochliobolus miyabeanus Cm*TG12bL2 assembly using Velvet. The white arrow indicates average weighted coverage (76.56X) of the assembly. The black arrow indicates the cut-off value (38.28X). These two values were used for optimizing the final *Cm*TG12bL2 assembly.
(DOCX)

S3 Fig Genome-wide DotPlot of *Cochliobolus miyabeanus* TG12bL2 and *C*. *miyabeanus* WK-1C. Ordered scaffolds of both species were used to build a dotplot matrix in Gepard software that uses suffix array data for heuristic dotplot computation. Minimum word length = 10.
(DOCX)

S4 Fig Alignment of effectors from *Cladosporium fulvum* with putative homologous fungal proteins. A. Multiple alignment of proteins with homology to Cf_Ecp2 = *Cladosporium fulvum*, Ch = *C*. *heterostrophus*; COCHEDRAFT_1130950_(gi|452001939), Cs = *C*. *sativus*; COCSADRAFT_196914_(gi|451853714), Cm = *C*. *miyabeanus* TG12bL2; CM_6804, and Fg = *Fusarium graminearum* (XP391494). B. Pairwise alignment of the Cf_Ecp6 and CM_2799 protein from *C*. *miyabeanus* TG12Lb2. Alignments were done with ClustalW (http://www.genome.jp/tools/clustalw/) with a dynamic programming method and using a default Blosum (for proteins) matrix, with gap penalties of 10 and gap extension penalties of 0.05. Identical amino acid residues are indicated with asterisks and highlighted in light gray, cysteines are highlighted in dark gray and LysM domains according to Cf_Ecp6 are indicated by continuous lines.
(DOCX)

S5 Fig Phylogenetic tree of adenylation binding domains of NRPS proteins of *Cochliobolus* species and other Dothediomycetes. Neighbor joining analysis of adenylation domains (AMPs) sequences of NRPS proteins (Left): I. AMPs of conserved NRPS proteins across *Cochliobolus* species (Red); II. AMPs of NRPS proteins with less degree of conservation in *Cochliobolus* species (Blue). III. AMPs of NRPS proteins with discontinuous distribution in *Cochliobolus* species (NRPS expanded group). IV. Outgroup set: Related adenylation modules: Long Chain Fatty Acid ligases (LCFA), Acyl-CoA synthetases (Acyl-CoA-Synth), Ochratoxin synthetases (ochratoxins), Acyl-CoA ligase (CPS1). ChetC4: *Cochliobolus heterostrophus* C4, ChetC5v2.0: *C*. *heterostrophus* C5, Cmiya: *C*. *miyabeanus* WK-1C, and Cmwr: *C*. *miyabeanus* TG12bL2. Other sequences are described in [40]. Blow-up clusters (Center): Main clusters harboring AMP domains related to Ch_NPS11 and GliP (gliotoxin) and SirP (sirodesmin) proteins sequences and of *Cm*TG12bL2 NPS1/NPS3/NPS13 expanded protein. Inset (Right): Graphic representation of AMP domains belonging to *C*. *heterostrophus* NPS3 and NPS13 (blue) and to *C*. *miyabeanus* NPS1/NPS3/NPS13 expanded protein (green).
(PDF)

S6 Fig Functional annotation of *Cochliobolus miyabeanus* TG12bL2 transcripts at 48 hours after inoculation of wildrice leaves. The sequence distributions by GO terms (level 2) of the fungal transcripts were done with Blast2Go software. A. *Biological processes*: 1. immune system process (GO:0002376), 2.single-organism process (GO:0044699), 3. response to stimulus (GO:0050896), 4. biological adhesion (GO:0022610), 5. cellular process (GO:0009987), 6. metabolic process (GO:0008152), 7. rhythmic process (GO:0048511), 8. cellular component organization or biogenesis (GO:0071840), 9. developmental process (GO:0032502), 10. reproduction (GO:0000003), 11. biological regulation (GO:0065007), 12. growth (GO:0040007), 13. locomotion (GO:0040011), 14. multi-organism process (GO:0051704), 15. localization (GO:0051179), 16. multicellular organismal process (GO:0032501), 17. signaling (GO:0023052). B. *Cellular component*: 1. extracellular region (GO:0005576), 2. nucleoid (GO:0009295), 3. symplast (GO:0055044), 4. synapse (GO:0045202), 5. virion (GO:0019012), 6. membrane (GO:0016020), 7. cell (GO:0005623), 8. organelle (GO:0043226), 9. macromolecular complex (GO:0032991), 10. membrane-enclosed lumen (GO:0031974), 11. cell junction (GO:0030054). C. *Molecular function*: 1. receptor activity (GO:0004872), 2. molecular transducer activity (GO:0060089), 3. protein binding transcription factor activity (GO:0000988), 4. nucleic acid binding transcription factor activity (GO:0001071), 5. structural molecule activity (GO:0005198), 6. electron carrier activity (GO:0009055), 7. antioxidant activity (GO:0016209), 8. enzyme regulator activity (GO:0030234), 9. binding (GO:0005488), 10. nutrient reservoir activity (GO:0045735), 11. catalytic activity (GO:0003824), 12. transporter activity (GO:0005215).
(TIF)

S7 Fig Relative expression of *Cm*TG12Lb2 selected genes by qRT-PCR at 24 h and 48 h after inoculation and transcriptome expression levels at 48 h after inoculation. A. The vertical axis shows the log _2_ of relative quantification (fold change) for each of the selected genes. The horizontal axis indicates two time points of sample collection after *Cm*TG12Lb2 inoculation (24 h and 48 h). The reference gene expression was set to 0 (Log_2_ (1). Gene expression was normalized with glyceraldehyde-3-phosphate dehydrogenase expression. B. Transcript abundance (RPKM Log_2_ transformed values) of six genes at 48 h after inoculation. Abreviations: *Ecp*6 = *Cladosporium fulvum Ecp*6 homolog, CYP53 = Cytochrome P450 CYP53, SH = salicylate hydroxylate, β-1,4-EG = β-1,4-endoglucanase, β-1,4-EX = β-1,4-endoxylanase, β-1,4-EG = β-1,4-glucosidase.
(TIF)

S1 Table Basic statistics and quality measures of the *Cochliobolus miyabeanus* TG12bL2 sequencing process. ^a^ The overall % GC of all bases in the sequences followed a normal distribution overlapping the theoretical distribution. Consistently, there were no overrepresented sequences or *k*-mers. ^b^ Quality scores across all bases sequenced. ^c^ Percentage of duplicated sequences relative to unique sequences indicating that some sequences had 10 or more duplicates.
(DOCX)

S2 Table Best hits of *C*. *miyabeanus* TG12bL2 genes to the CEGMA (Core Eukaryotic Gene Mapping Approach) gene list from six eukaryotic genomes: *Homo sapiens* (Hs), *Drosophila melanogaster*, *Caenorhabditis elegans* (CE), *Arabidopsis thaliana* (At), *Saccharomyces cerevisiae* (Y), and *Schizosaccharomyces pombe* (SP). Function. *Cellular processes and signaling*: M: Cell wall/membrane/envelope biogenesis, O Posttranslational modification, protein turnover, chaperones, T: Signal transduction mechanisms, U: Intracellular trafficking, secretion, and vesicular transport, V: Defense mechanisms, Y: Nuclear structure, Z: Cytoskeleton. *Information storage and processing*: A: RNA processing and modification, B: Chromatin structure and dynamics, J: Translation, ribosomal structure and biogenesis, K: Transcription, L: Replication, recombination and repair. *Metabolism*: C: Energy, D: Cell cycle control, cell division, chromosome partitioning, E: Amino acid transport and metabolism, G: Carbohydrate transport and metabolism, H: Coenzyme transport and metabolism, I: Lipid transport and metabolism, P: Inorganic ion transport and metabolism, Q: Secondary metabolites biosynthesis, transport and catabolism. *Poorly characterized*: R: General function prediction only, S: Function unknown.
(XLSX)

S3 Table Comparison of small secreted proteins in *C*. *miyabeanus Cm*TG12bL2 to those reported for eighteen Dothidiomycete genomes. (a) HMMER using hmmscan algorithm to compare protein sequences against collections of profiles (only Pfam protein family was used). Proteins with transmembrane regions as detected by *Phobius* were removed from the analysis. (b) Values of HCSSPs calculated as in [28] (as least twice the general average of cysteines in the *Cm*TG12bL2 proteome), and (c) as in [24] (over 2% of cysteines). All SSPs were under 200 amino acids in length.
(XLSX)

S4 Table Pfam domains identified in *C*. *miyabeanus* TG12bL2 small secreted proteins. Pfam domains were identified using HMMER software (http://hmmer.janelia.org) with the algorithm/program hmmscan and a gathering cut off to compare protein sequences against collections of profiles (only Pfam protein family was used). Proteins with transmembrane regions as detected by *Phobius* were removed from the analysis.
(XLSX)

S5 Table Distribution of carbohydrate active enzyme families in 12 Ascomycete genomes. GH: Glycosyl hydrolases; ^(a)^: Indicates GH5 Sub family; GT: glycosyl transferases, PL: polysaccharide lyases, and CE: carbohydrate esterases, CBM: carbohydrate binding modules.
(XLSX)

S6 Table Identification of CAZyme in *Cm*TG12bL2 genome associated to degradation of cellulose, hemicellulose, and pectin. (XLSX)

S7 Table *Cochliobolus miyabeanus* TG12bL2 predicted P450 monooxygenases. Fungal Cytochrome P450 Database (FCPD; http://p450.riceblast.snu.ac.kr/download.php).
(XLSX)

S1 Text CAZymes supplementary information. (DOCX)

## References

[1] Datnoff LE, Raid RN, Snyder GH, Jones DB. Effect of calcium silicate on blast and brown spot intensities and yields of rice. Plant Dis. 1991; 75: 729–732.

[2] Farr DF, Bills GF, Chamuris GP, Rossman AY. Fungi on plants and plant products in the United States. APS PRESS: St. Paul; 1989.

[3] Johnson DR, Percich JA. Wildrice domestication, fungal brown spot disease and the future of commercial production in Minnesota. Plant Dis. 1992; 76: 1193–1198.

[4] Krupinsky JM, Berdahl JD, Schoch CL, Rossman AY. Leaf spot on switch grass (*Panicum virgatum*), symptoms of a new disease caused by *Bipolaris oryzae*. Can J Plant Pathol. 2004; 26: 371–378.

[5] Ou SH. Rice Diseases. 2nd. Ed Commonwealth Mycological Institute, Kew, England 1985.

[6] Xu X, Walters C, Antolin ML, Alexander ML, Lutz S, Ge S, et al Phylogeny and biogeography of eastern Asian-North American disjunct wild-rice genus (*Zizania* L. *Poaceae*). Mol Phylogenet Evol. 2009; 55: 1008–1017. 10.1016/j.ympev.2009.11.018 19944174

[7] Oelke EA. Saga of the grain: A tribute to Minnesota cultivated wild rice growers. Schreiner R., ed. Hobar Publications, Lakeville, MN; 2007.

[8] Kohls CL, Percich JA, Huot CM. Wildrice yield losses associated with growth-stage-specific fungal brown spot epidemics. Plant Dis. 1987; 71: 419–422.

[9] Nyvall RF, Percich JA. Development of fungal brown spot and spot blotch on cultivated wild rice in Minnesota. Plant Dis. 1999; 83: 936–938. 10.1094/PDIS.1999.83.10.936 30841076

[10] Moffatt AM. Evaluation of various grasses as hosts for *Bipolaris oryzae*, causal organism of fungal brown spot on cultivated wildrice in Minnesota. MS Thesis, University of Minnesota. 1998.

[11] Porter R. Variety trial results: Wildrice Minnesota Agricultural Experiment Station. University of Minnesota; 2010.

[12] Wolpert TJ, Macko V, Acklin W, Jaun B, Seibl J, Meili J, et al Structure of victorin C, the major host-selective toxin from *Cochliobolus victoriae*. Experientia. 1985; 41: 1524–1529.

[13] Lorang J, Kidarsa T, Bradford CS, Gilbert B, Curtis M, Tzeng SC, et al Tricking the guard: exploiting plant defense for disease susceptibility. Science. 2012; 338: 659–662. 10.1126/science.1226743 23087001 PMC4125361

[14] Scott-Craig JS, Panaccione DG, Pocard JA, Walton JD. The cyclic peptide synthetase catalyzing HC-toxin production in the filamentous fungus *Cochliobolus carbonum* is encoded by a 15.7-kilobase open reading frame. J Biol Chem. 1992; 267: 26044–26049. 1281482

[15] Pitkin JW, Panaccione DG, Walton JD. A putative cyclic peptide efflux pump encoded by the TOXA gene of the plant-pathogenic fungus *Cochliobolus carbonum*. Microbiology. 1996; 142: 1557–1565. 8704997 10.1099/13500872-142-6-1557

[16] Ahn JH, Walton JD. A fatty acid synthase gene in *Cochliobolus carbonum* required for production of HC-toxin, cyclo (D-prolyl-L-alanyl-D-alanyl-L-2-amino-9, 10-epoxi-8-oxodecanoyl). Mol Plant Microbe Interact. 1997; 10: 207–214. 9057326 10.1094/MPMI.1997.10.2.207

[17] Cheng YQ, Ahn JH, Walton JD. A putative branched-chain-amino-acid transaminase gene required for HC-toxin biosynthesis and pathogenicity in *Cochliobolus carbonum*. Microbiology. 1999; 145: 3539–3546. 10627051 10.1099/00221287-145-12-3539

[18] Cheng YQ Walton JD. A eukaryotic alanine racemase gene involved in cyclic peptide biosynthesis. J Biol Chem. 2000; 275: 4906–4911. 10671527 10.1074/jbc.275.7.4906

[19] Ahn JH, Walton JD. Regulation of cyclic peptide biosynthesis and pathogenicity in *Cochliobolus carbonum* by TOXEp, a novel protein with a bZIP basic DNA-binding motif and four ankyrin repeats. Mol Gen Genet. 1998; 260: 462–469. 9894916 10.1007/pl00008632

[20] Turgeon BG, Baker SE. Genetic and genomic dissection of the *Cochliobolus heterostrophus* Tox1 locus controlling biosynthesis of the polyketide virulence factor T-toxin. Adv Genet. 2007; 57: 219–261. 17352906 10.1016/S0065-2660(06)57006-3

[21] Yang G, Rose MS, Turgeon GB, Yoder OC. A polyketide synthase is required for funga1 virulence and production of the polyketide T-toxin. Plant Cell. 1996; 8: 2139–2150. 8953776 10.1105/tpc.8.11.2139 PMC161341

[22] Baker SE, Kroken S, Inderbitzin P, Asvarak T, Li BY, Shi L, et al Two polyketide synthase-encoding genes are required for biosynthesis of the polyketide virulence factor, T-toxin, by *Cochliobolus heterostrophus*. Mol Plant Microbe Interact. 2006; 19: 139–149. 16529376 10.1094/MPMI-19-0139

[23] Rose MS, Yun SH, Asvarak T, Lu SW, Yoder OC, Turgeon BG. A decarboxylase encoded at the *Cochliobolus heterostrophus* translocation-associated Tox1B locus is required for polyketide (T-toxin) biosynthesis and high virulence on T-cytoplasm maize. Mol Plant Microbe Interact. 2002; 15: 883–893. 12236595 10.1094/MPMI.2002.15.9.883

[24] Condon BJ, Leng Y, Wu D, Bushley KE, Ohm RA, Otillar R, et al Comparative genome structure, secondary metabolite, and effector coding capacity across *Cochliobolus* pathogens. PLOS Genet. 2012; 9: 1–29. 10.1371/journal.pgen.1003233 PMC3554632 23357949

[25] Xiao JZ, Tsuda M, Doke N, Nishimura S. Phytotoxins produced by germinating spores of *Bipolaris oryzae*. Phytopathology. 1990; 81: 58–64.

[26] Au TK, Chick WS, Leung PC. The biology of ophiobolins. Life Sci. 2000; 67: 733–742. 10968403 10.1016/s0024-3205(00)00668-8

[27] Fukushima Y, Sakagami Y, Marumo S. Beta-Glucan biosynthesis inhibitors isolated from fungi as hyphal malformation inducer. Bioorg Med Chem Lett. 1993; 3: 1219–1222.

[28] Ohm R, Feau N, Henrissat B, Schoch CL, Horwitz BA, Barry KW, et al Diverse lifestyles and strategies of plant pathogenesis encoded in the genomes of eighteen dothideomycetes fungi. PLoS Pathog 2012; 8 (12): e1003037 10.1371/journal.ppat.1003037 23236275 PMC3516569

[29] Castell-Miller CV, Samac DA. Population genetic structure, gene flow and recombination of *Cochliobolus miyabeanus* in cultivated wildrice (*Zizania palustris* L.). Plant Pathol. 2012; 61: 905–914.

[30] Yoder OC. *Cochliobolus heterostrophus*, cause of southern corn leaf blight. Adv Plant Pathol. 1988; 6: 93–112.

[31] Raeder U, Broda P. Rapid preparation of DNA from filamentous fungi. Lett Appl Microbiol. 1985; 1: 17–20.

[32] Zerbino DR, Birney E. Velvet: Algorithms for *de novo* short read assembly using de Bruijn graphs. Genome Res. 2008; 18: 821–829. 10.1101/gr.074492.107 18349386 PMC2336801

[33] Zerbino DR. Using the Velvet de novo assembler for short-read sequencing technologies. Curr Protoc Bioinform. 2010; 11: 11.5.1–11.5.12. 10.1002/0471250953.bi1105s31 PMC2952100 20836074

[34] Ter-Hovhannishyan V, Lomsadze A, Chernoff YO, Borodovsky M. Gene prediction in novel fungal genomes using an *ab initio* algorithm with unsupervised training. Genome Res. 2008; 18: 1979–1990. 10.1101/gr.081612.108 18757608 PMC2593577

[35] Brendtsen J, Nielsen H, von Heijne G, Brunak S. Improved prediction of signal peptides: SignalP3.0. J Mol Biol. 2004; 340: 783–795. 15223320 10.1016/j.jmb.2004.05.028

[36] Parra G, Bradnam K, Korf I. CEGMA: a pipeline to accurately annotate core genes in eukaryotic genomes. Bioinformatics. 2007; 23: 1061–1067. 17332020 10.1093/bioinformatics/btm071

[37] Krumsiek J, Arnold R, Tattei T. Gepard: a rapid and sensitive tool for creating dotplots on genome scale. Bioinformatics. 2007; 23: 1026–1028. 17309896 10.1093/bioinformatics/btm039

[38] Lombard V, Golaconda Ramulu H, Drula E, Coutinho PM, Henrissat B. The carbohydrate-active enzymes database (CAZy) in 2013. Nucleic Acids Res. 2014; 42: D490–D495. 10.1093/nar/gkt1178 24270786 PMC3965031

[39] Cantarel BL, Coutinho PM, Rancurel C, Bernard T, Lombard V, Henrissat B. The Carbohydrate-Active EnZymes database (CAZy): an expert resource for glycogenomics. Nucleic Acids Res. 2009; 37: D233–D238. 10.1093/nar/gkn663 18838391 PMC2686590

[40] Bushley KE, Turgeon BG. Phylogenomics reveals subfamilies of fungal nonribosomal peptide synthetases and their evolutionary relationships. BMC Evol Biol. 2010; 10: 1471–2148. 10.1186/1471-2148-10-26 PMC2823734 20100353

[41] Kroken S, Glass NL, Taylor JW, Yoder OC, Turgeon BG. Phylogenomic analysis of type I polyketide synthase genes in pathogenic and saprobic ascomycetes. Proc Natl Acad Sci USA. 2003; 100: 15670–15675. 14676319 10.1073/pnas.2532165100 PMC307626

[42] Lee B-N, Kroken S, Chou DY-T, Robbertse B, Yoder OC, Turgeon BG. Functional analysis of all non-ribosomal peptide synthetases in *Cochliobolus heterostrophus* reveals a factor, NPS6, involved in virulence and resistance to oxidative stress. Eukaryot Cell. 2005; 4: 545–555. 15755917 10.1128/EC.4.3.545-555.2005 PMC1087798

[43] Gutierrez-Gonzalez JJ, Tu ZJ, Garvin DF. Analysis and annotation of the hexaploid oat seed transcriptome. BMC Genomics. 2013; 14: 471–487. 10.1186/1471-2164-14-471 23845136 PMC3720263

[44] Trapnell C, Pachter L, Salzberg SL. TopHat: discovering splice junctions with RNA-Seq. Bioinformatics. 2009; 25: 1105–1111. 10.1093/bioinformatics/btp120 19289445 PMC2672628

[45] Trapnell C, Williams BA, Pertea G, Mortazavi A, Kwan G, van Baren MJ, et al Transcript assembly and quantification by RNA-Seq reveals unannotated transcripts and isoform switching during cell differentiation. Nat Biotechnol. 2010; 28: 511–515. 10.1038/nbt.1621 20436464 PMC3146043

[46] Mortazavi A, Williams BA, McCue K, Schaeffer L, Wold B. Mapping and quantifying mammalian transcriptomes by RNA-Seq. Nat Methods. 2008; 5:621–628. 10.1038/nmeth.1226 18516045 PMC13303166

[47] Al-Shahrour F, Diaz-Uriarte R, Dopazo J. FatiGO: a web tool for finding significant associations of Gene Ontology terms with groups of genes. Bioinformatics. 2004; 20:578–580. 14990455 10.1093/bioinformatics/btg455

[48] Ganem S, Lu S-W, Lee B-N, Chou DY-T, Hadar R, Turgeon BG, et al G-protein β subunit of *Cochliobolus heterostrophus* involved in virulence, asexual and sexual reproductive ability, and morphogenesis. Eukaryot Cell. 2004; 3: 1653–1663. 15590838 10.1128/EC.3.6.1653-1663.2004 PMC539015

[49] Lu S-W, Kroken S, Lee B-N, Robbertse B, Churchill ACL, Yoder OC, et al A novel class of gene controlling virulence in plant pathogenic ascomycete fungi. Proc Natl Acad Sci USA. 2003; 100: 5980–5985. 12730371 10.1073/pnas.0931375100 PMC156312

[50] Baidyaroy D, Brosch G, Ahn J-H, Graessle S, Wegener S, Tonukari NJ, et al A gene related to yeast *hos2* histone deacetylase affects extracellular depolymerase expression and virulence in a plant pathogenic fungus. Plant Cell. 2001; 13: 1609–1624. 11449054 10.1105/TPC.010168 PMC139552

[51] Trojer P, Brandtner EM, Brosch G, Loidl P, Galehr J, Linzmaier R, et al Histone deacetylases in fungi: novel members, new facts. Nucleic Acids Res. 2003; 31: 3971–3981. 12853613 10.1093/nar/gkg473 PMC167634

[52] Tonukari NJ, Scott-Craig JS, Walton JD. The *Cochliobolus carbonum* SNF1 gene is required for cell-wall enzyme expression and virulence on maize. Plant Cell. 2000; 12: 237–247. 10662860 10.1105/tpc.12.2.237 PMC139761

[53] Tonukari NJ, Scott-Craig JS, Walton JD. Isolation of the carbon catabolite repressor (CREA) gene from the plant-pathogenic fungus *Cochliobolus carbonum*. DNA Sequence. 2003; 14: 103–107. 12825351 10.1080/1042517031000073727

[54] Moriwaki A, Kihara J, Mori CC, Arase S. A MAP kinase gene, BMK1, is required for conidiation and pathogenicity in the rice leaf spot pathogen *Bipolaris oryzae*. Microbiol Res. 2007; 162: 108–114. 16546358 10.1016/j.micres.2006.01.014

[55] Oide S, Moeder W, Krasnoff SB, Gibson DM, Haas H, Yoshioka K, et al *NPS6*, encoding a non-ribosomal peptide synthetase involved in siderophore-mediated iron metabolism, is a conserved virulence determinant of plant pathogenic ascomycetes. Plant Cell. 2006; 18: 2836–2853. 17056706 10.1105/tpc.106.045633 PMC1626607

[56] Lev S, Hadar R, Amedeo P, Baker SE, Yoder OC, Horwitz BA. Activation of an AP1-like transcription factor of the maize pathogen *Cochliobolus heterostrophus* in response to oxidative stress and plant signals. Eukaryot Cell. 2005; 4: 443–454. 15701806 10.1128/EC.4.2.443-454.2005 PMC549334

[57] Scott-Craig JS, Panaccione DG, Cervone F, Walton JD. Endopolygalacturonase is not required for pathogenicity of *Cochliobolus carbonum* on maize. Plant Cell. 1990; 2: 1191–2000. 2152162 10.1105/tpc.2.12.1191 PMC159966

[58] Scott-Craig JS, Cheng YQ, Cervone F, De Lorenzo G, Pitkin JW, Walton JD. Targeted mutants of *Cochliobolus carbonum* lacking the two major extracellular polygalacturonases. Appl Environ Microbiol. 1998; 64: 1497–503. 9546185 10.1128/aem.64.4.1497-1503.1998 PMC106176

[59] Apel-Birkhold PC, Walton JD. Cloning, disruption, and expression of two endo-beta 1, 4-xylanase genes, XYL2 and XYL3, from *Cochliobolus carbonum*. Appl Environ Microbiol. 1996; 62: 4129–4135. 8900004 10.1128/aem.62.11.4129-4135.1996 PMC168235

[60] Ahn JH, Sposato P, Kim SI, Walton JD. Molecular cloning and characterization of cel2 from the fungus *Cochlibolus carbonum*. Biosci Biotechnol Biochem. 2001; 65: 1406–1411. 11471744 10.1271/bbb.65.1406

[61] Murphy JM, Walton JD. Three extracellular proteases from *Cochliobolus carbonum*: cloning and targeted disruption of ALP1. Mol Plant Microbe Interact. 1996; 9: 290–297. 8634479 10.1094/mpmi-9-0290

[62] Bayry J, Aimanianda V, Guijarro JI, Sunde M, Latgé JP. Hydrophobins: unique fungal proteins. PLoS Pathog. 2012; 8: 1–4. 10.1371/journal.ppat.1002700 PMC3364958 22693445

[63] Kulkarni RD, Kelkar HS, Dean RA. An eight-cysteine-containing CFEM domain unique to a group of fungal membrane proteins. Trends Biochem Sci. 2003; 28: 118–121. 12633989 10.1016/S0968-0004(03)00025-2

[64] Pazzaglia L, Seidl-Seibothb V, Barsottinic M, Vargas WA, Scalae A, Mukherjee PK. Cerato-platanins: Elicitors and effectors. Plant Sci. 2014; 228: 79–87. 10.1016/j.plantsci.2014.02.009 25438788

[65] Laugé R, Joosten MHAJ, van den Ackerveken GFJM, van den Broek HWJ, de Wit PJGM. The in planta-produced extracellular proteins ECP1 and ECP2 of *Cladosporium fulvum* are virulence factors. Mol Plant Microbe Interact. 1997; 10: 725–734.

[66] Stergiopoulos I, van den Burg HA, Ölmen B, Beenen HG, van Liere S, Kema GHJ, et al Tomato Cf resistance proteins mediate recognition of cognate homologous effectors from fungi pathogenic on dicots and monocots. Proc Natl Acad Sci USA. 2010; 107: 7610–7615. 10.1073/pnas.1002910107 20368413 PMC2867746

[67] Bolton MD, van Esse HP, Vossen JH, de Jonge R, Stergiopoulos I, Stulemeijer IJ, et al The novel *Cladosporium fulvum* lysin motif effector Ecp6 is a virulence factor with orthologues in other fungal species. Mol Microbiol. 2008; 69: 119–136. 10.1111/j.1365-2958.2008.06270.x 18452583

[68] de Jonge R, Thomma BPHJ. Fungal LysM effectors: extinguishers of host immunity? Trends Microbiol. 2009; 17: 151–157. 10.1016/j.tim.2009.01.002 19299132

[69] de Jonge R, van Esse HP, Kombrink A, Shinya T, Desaki Y, Bours R, et al Conserved fungal LysM effector Ecp6 prevents chitin-triggered immunity in plants. Science. 2010; 329: 953–955. 10.1126/science.1190859 20724636

[70] Bhadauria V, Banniza S, Vandenberg A, Selvaraj G, Wei Y. EST mining identifies proteins putatively secreted by the anthracnose pathogen *Colletotrichum truncatum*. BMC Genomics 2011; 12: 1–16. 10.1186/1471-2164-12-327 PMC3149586 21699715

[71] Marshall R, Kombrink A, Motteram J, Loza-Reyes E, Lucas J, Hammond-Kosack KE, et al Analysis of two *in planta* expressed LysM effector homologues from the fungus *Mycosphaerella graminicola* reveals novel functional properties and varying contributions to virulence on wheat. Plant Physiol. 2011; 156: 756–769. 10.1104/pp.111.176347 21467214 PMC3177273

[72] Kleemann J, Rincon-Rivera LJ, Takahara H, Neumann U, van Themaat EVL, van der Does HC, et al Sequential delivery of host-induced virulence effectors by appressoria and intracellular hyphae of the phytopathogen *Colletotrichum higginsianum*. PLoS Pathog. 2012; 8: 1–15. 10.1371/journal.ppat.1002643 PMC3320591 22496661

[73] Qutob D, Kamoun S, Gijzen M. Expression of a *Phytophthora sojae* necrosis-inducing proteins occurs during transition from biotrophy to necrotrophy. Plant J. 2002; 32: 361–373. 12410814 10.1046/j.1365-313x.2002.01439.x

[74] Motteram J, Küfner I, Deller S, Brunner F, Hammond-Kosack KE, Nürnberger T, et al Molecular characterization and functional analysis of *MgNLP*, the sole NPP1 domain–containing protein, from the fungal wheat leaf pathogen *Mycosphaerella graminicola*. Mol Plant Microbe Interact. 2009; 22: 790–799. 10.1094/MPMI-22-7-0790 19522561

[75] Cuesta Arenas Y, Kalkman ERIC, Schouten A, Dieho M, Vredenbregt P, Uwumukiza B, et al Functional analysis and mode of action of phytotoxic Nep1-like proteins of *Botrytis cinerea*. Physiol Mol Plant Pathol. 2010; 74: 376–386.

[76] Levasseur A, Drula E, Lombard V, Coutinho PM, Henrissat B. Expansion of the enzymatic repertoire of the CAZy database to integrate auxiliary redox enzymes. Biotechnol Biofuels. 2013; 6: 41–54. 10.1186/1754-6834-6-41 23514094 PMC3620520

[77] van den Brink J, de Vries RP. Fungal enzyme sets for plant polysaccharide degradation. Appl Microbiol Biotechnol. 2011; 91: 1477–1492. 10.1007/s00253-011-3473-2 21785931 PMC3160556

[78] Burton RA, Fincher GB. (1,3;1,4)-Beta-D-Glucans in cell walls of the Poaceae, lower plants, and fungi: A tale of two linkages. Mol Plant. 2009; 2: 873–882. 10.1093/mp/ssp063 19825664

[79] Seidl V. Chitinases of filamentous fungi: a large group of diverse proteins with multiple physiological functions. Fungal Biol Rev. 2008; 22: 36–42.

[80] Klutts JS, Yoneda A, Reilly MC, Bose I, Doering T. Glycosyltransferases and their products: cryptococcal variations on fungal themes. FEMS Yeast Res. 2006; 6: 499–512. 16696646 10.1111/j.1567-1364.2006.00054.x

[81] Sexton AC, Minic Z, Cozijnsen AJ, Pedras MSC, Howlett BJ. Cloning, purification and characterisation of brassinin glucosyltransferase, a phytoalexin-detoxifying enzyme from the plant pathogen *Sclerotinia sclerotiorum*. Fungal Genet Biol. 2009; 46: 201–209. 10.1016/j.fgb.2008.10.014 19041410

[82] Hartl L, Zach S, Seidl-Seiboth V. Fungal chitinases: diversity, mechanistic properties and biotechnological potential. Appl Microbiol Biotechnol. 2012; 93: 533–543. 10.1007/s00253-011-3723-3 22134638 PMC3257436

[83] Boraston AB, Bolam DN, Gilbert HJ, Davies GJ. Carbohydrate-binding modules: fine-tuning polysaccharide recognition. Biochem J. 2004; 382: 769–781. 15214846 10.1042/BJ20040892 PMC1133952

[84] . Kubicek CP, Seidl V, Seiboth B. Plant cell wall and chitin degradation In: Borkovich K, Ebbole DJ, editors. Cellular and molecular biology of filamentous fungi. Washington DC: AMS Press; 2010 pp. 316–413.

[85] Varki A, Cummings RD, Aebi M, Packer NH, Seeberger PH, Esko JD, et al Symbol nomenclature for graphical representations of glycans. Glycobiology. 2015; 25:1323–1324. 10.1093/glycob/cwv091 26543186 PMC4643639

[86] Turgeon BG, Bushley KE. Secondary metabolism In: Borkovich KA, Ebbole DJ, editors. Cellular and molecular biology of filamentous fungi. Washington DC: AMS Press; 2010 pp. 376–395.

[87] Schwarzer D, Finking R, Marahiel MA. Nonribosomal peptides: from genes to products. Nat Prod Rep. 2003; 20: 275–287. 12828367 10.1039/b111145k

[88] Turgeon BG, Oide S, Bushley KE. Creating and screening *Cochliobolus heterostrophus* non-ribosomal peptide synthetase mutants. Mycol Res. 2008; 112: 200–206. 10.1016/j.mycres.2007.10.012 18280721

[89] Oide S, Krasnoff SB, Gibson DM, Turgeon BG. Intracellular siderophores are essential for ascomycete sexual development in heterothallic *Cochliobolus heterostrophus* and homothallic *Gibberella zeae*. Eukaryot Cell. 2007; 6: 1339–1353. 17601875 10.1128/EC.00111-07 PMC1951124

[90] Kim K-H, Cho Y, La Rota M, Cramer RA, Lawrence C. Functional analysis of the *Alternaria brassicicola* non-ribosomal peptide synthetase gene AbNPS2 reveals a role in conidial cell wall construction. Mol Plant Path. 2007; 8: 23–39. 20507476 10.1111/j.1364-3703.2006.00366.x

[91] Crešnar B, Petrič S. Cytochrome P450 enzymes in the fungal kingdom. Biochim Biophys Acta. 2011; 1814: 2 9–35. 10.1016/j.bbapap.2010.06.020 20619366

[92] Moktali V, Park J, Fedorova-Abrams ND, Park B, Choi J, Lee Y-H, et al Systematic and searchable classification of cytochrome P450 proteins encoded by fungal and oomycete genomes. BMC Genomics. 2012; 13: 525–537. 10.1186/1471-2164-13-525 23033934 PMC3505482

[93] Reimmann C, VanEtten HD. Cloning and characterization of the *PDA6-1* gene encoding a fungal cytochrome P-450 which detoxifies the phytoalexin pisatin from garden pea. Gene. 1994; 146: 221–226. 8076822 10.1016/0378-1119(94)90296-8

[94] Podobnik B, Stojan J, Lah L, Krasevec N, Seliskar M, Lanisnik Rizner T, et al CYP53A15 of *Cochliobolus lunatus*, a target for natural antifungal compounds. J Med Chem. 2008; 51: 3480–3486. 10.1021/jm800030e 18505250

[95] Mingot JM, Penalva MA, Fernandez-Canon M. Disruption of *phacA*, an *Aspergillus nidulans* gene encoding a novel cytochrome P450 monooxygenase catalyzing phenylacetate 2-hydroxylation, results in penicillin overproduction. J Biol Chem. 1999; 274: 14545–14550. 10329644 10.1074/jbc.274.21.14545

[96] Ferrer-Sevillano F, Fernandez-Canon JM. Novel *phacB*-encoded cytochrome P450 monooxygenase from *Aspergillus nidulans* with 3-hydroxyphenylacetate 6-hydroxylase and 3,4-dihydroxyphenylacetate 6-hydroxylase activities. Eukaryot Cell. 2007; 6: 514–520. 17189487 10.1128/EC.00226-06 PMC1828918

[97] Del Sorbo G, Schoonbeek H, De Waard MA. Fungal transporters involved in efflux of natural toxic compounds and fungicides. Fungal Genet Biol. 2000; 30:1–15. 10955904 10.1006/fgbi.2000.1206

[98] Jørgensen TR, vanKuyk PA, Poulsen BR, Ruijter GJG, Visser J, Iversen JJL. Glucose uptake and growth of glucose-limited chemostat cultures of *Aspergillus niger* and a disruptant lacking MstA, a high-affinity glucose transporter Microbiology. 2007; 153: 1963–1973. 17526853 10.1099/mic.0.2006/005090-0

[99] Leandro MJ, Gonçalves P, Spencer-Martins I. Two glucose/xylose transporter genes from the yeast *Candida intermedia*: first molecular characterization of a yeast xylose–H+ symporter. Biochem. J. 2006; 395: 543–549. 16402921 10.1042/BJ20051465 PMC1462686

[100] Alarco A-M, Balan I, Talibi D, Mainville N, Raymond M. AP1-mediated multidrug resistance in *Saccharomyces cerevisiae* requires *FLR1* encoding a transporter of the major facilitator superfamily. J Biol Chem. 1997; 272: 19304–19313. 9235926 10.1074/jbc.272.31.19304

[101] Llorente B, Dujon B. Transcriptional regulation of the *Saccharomyces cerevisiae* DAL5 gene family and identification of the high affinity nicotinic acid permease TNA1 (YGR260w). FEBS Letters. 2000; 475: 237–241. 10869563 10.1016/s0014-5793(00)01698-7

[102] de Waard MA, Andrade AC, Hayashi K, Schoonbeek H, Stergiopoulos I, Zwiers L. Impact of fungal drug transporters on fungicide sensitivity, multidrug resistance and virulence. Pest Manag Sci. 2006; 62: 195–207. 16475240 10.1002/ps.1150

[103] Coleman JJ, Mylonakis E. Efflux in fungi: La pièce de résistance. PLoS Pathog. 2009; 5: 1–7. 10.1371/journal.ppat.1000486 PMC2695561 19557154

[104] Bissinger PH, Kuchled K. Molecular cloning and expression of the *Saccharomyces cerevisiae STSl*. J Chem. 1994; 269: 4180–4186. 8307980

[105] Kim JY, Wu J, Kwon SJ, Oh H, Lee SE, Kim SG, et al Proteomics of rice and *Cochliobolus miyabeanus* fungal interaction: insight into proteins at intracellular and extracellular spaces. Proteomics. 2014, 14:2307–2018. 10.1002/pmic.201400066 25047395

[106] Condon BJ, Wu D, Krasevec N, Horwitz BA, Turgeon BG. Comparative genomics of *Cochliobolus* phytopathogens In: Dean R, Lichens-Park A, Kole C, editors. Genomics of plant-associated fungi and oomycetes.Berlin: Springer; 2013 pp. 41–67.

[107] Lowe RGT, Howlett BJ. Indifferent, Affectionate, or Deceitful: lifestyles and secretomes of fungi. PLoS Pathog. 2012; 8: 1–3. 10.1371/journal.ppat.1002515 PMC3291654 22396640

[108] Boddi S, Comparini C, Calamassi R, Pazzagli L, Cappugi G, Scala A. Cerato-platanin protein is located in the cell walls of ascospores, conidia and hyphae of *Ceratocystis fimbriata* f. sp. *platani*. FEMS Microbiol Lett. 2004; 233: 341–346. 15063505 10.1016/j.femsle.2004.03.001

[109] Pazzagli L, Cappugi G, Manao G, Camici G, Santini A, Scala A. Purification, characterization, and amino acid sequence of cerato-platanin, a new phytotoxic protein from *Ceratocystis fimbriata* f. sp. *platani*. J Biol Chem. 1999; 274: 24959–24964. 10455173 10.1074/jbc.274.35.24959

[110] Baccelli I. Cerato-platanin family proteins: one function for multiple biological roles? Front Plant Sci. 2005; 5: 1–4. 10.3389/fpls.2014.00769 PMC4284994 25610450

[111] Kaku H, Nishizawa Y, Ishii-Minami N, Akimoto-Tomiyama C, Dohmae N, Takio K, et al Plant cells recognize chitin fragments for defense signaling through a plasma membrane receptor. Proc Natl Acad Sci USA. 2006; 103: 11086–11091. 16829581 10.1073/pnas.0508882103 PMC1636686

[112] Shimizu T, Nakano T, Takamizawa D, Desaki Y, Ishii-Minami N, Nishizawa Y, et al Two LysM receptor molecules, CEBiP and OsCERK1, cooperatively regulate chitin elicitor signaling in rice. Plant J. 2010; 64: 204–214. 10.1111/j.1365-313X.2010.04324.x 21070404 PMC2996852

[113] Vidhyasekaran P, Borromeo ES, Mew TW. *Helminthosporium oryzae* toxin suppresses phenol metabolism in rice plants and aids pathogen colonization. Physiol and Mol Plant Pathol. 1992; 41: 307–315.

[114] Degani O. Gene expression modulation of two biosynthesis pathways via signal transduction in *Cochliobolus heterostrophus*. Adv Biosci Biotechnol. 2014; 5: 340–352.

[115] Brown NA, Antoniw J, Hammond-Kosack KE. The predicted secretome of the plant pathogenic fungus *Fusarium graminearum*: a refined comparative analysis. PLoS ONE. 2012; 7:1–14. 10.1371/journal.pone.0033731 PMC3320895 22493673

[116] Yazawa T, Kawahigashi H, Matsumoto T, Mizuno H. Simultaneous transcriptome analysis of sorghum and *Bipolaris sorghicola* by using RNA-seq in combination with *De Novo* transcriptome assembly. PLoS ONE. 2013; 8:1–13. 10.1371/journal.pone.0062460 PMC3640049 23638091

