# Supplementary material for: Genome Assembly of the Fungus Cochliobolus miyabeanus, and Transcriptome Analysis during Early Stages of Infection on American Wildrice (Zizania palustris L.)
Source: PLoS One. 2016 Jun 2;11(6):e0154122. doi: 10.1371/journal.pone.0154122 (PMC4890743; doi:10.1371/journal.pone.0154122)
Supplement: S2 Fig — The white arrow indicates average weighted coverage (76.56X) of the assembly. The black arrow indicates the cut-off value (38.28X). These two values were used for optimizing the final CmTG12bL2 assembly. (DOCX) [file pone.0154122.s002.docx]

Average – weighted coverage
