# Supplementary figures and images for: Genome Assembly of the Fungus Cochliobolus miyabeanus, and Transcriptome Analysis during Early Stages of Infection on American Wildrice (Zizania palustris L.)
Source: PLoS One. 2016 Jun 2;11(6):e0154122. doi: 10.1371/journal.pone.0154122 (PMC4890743; doi:10.1371/journal.pone.0154122)

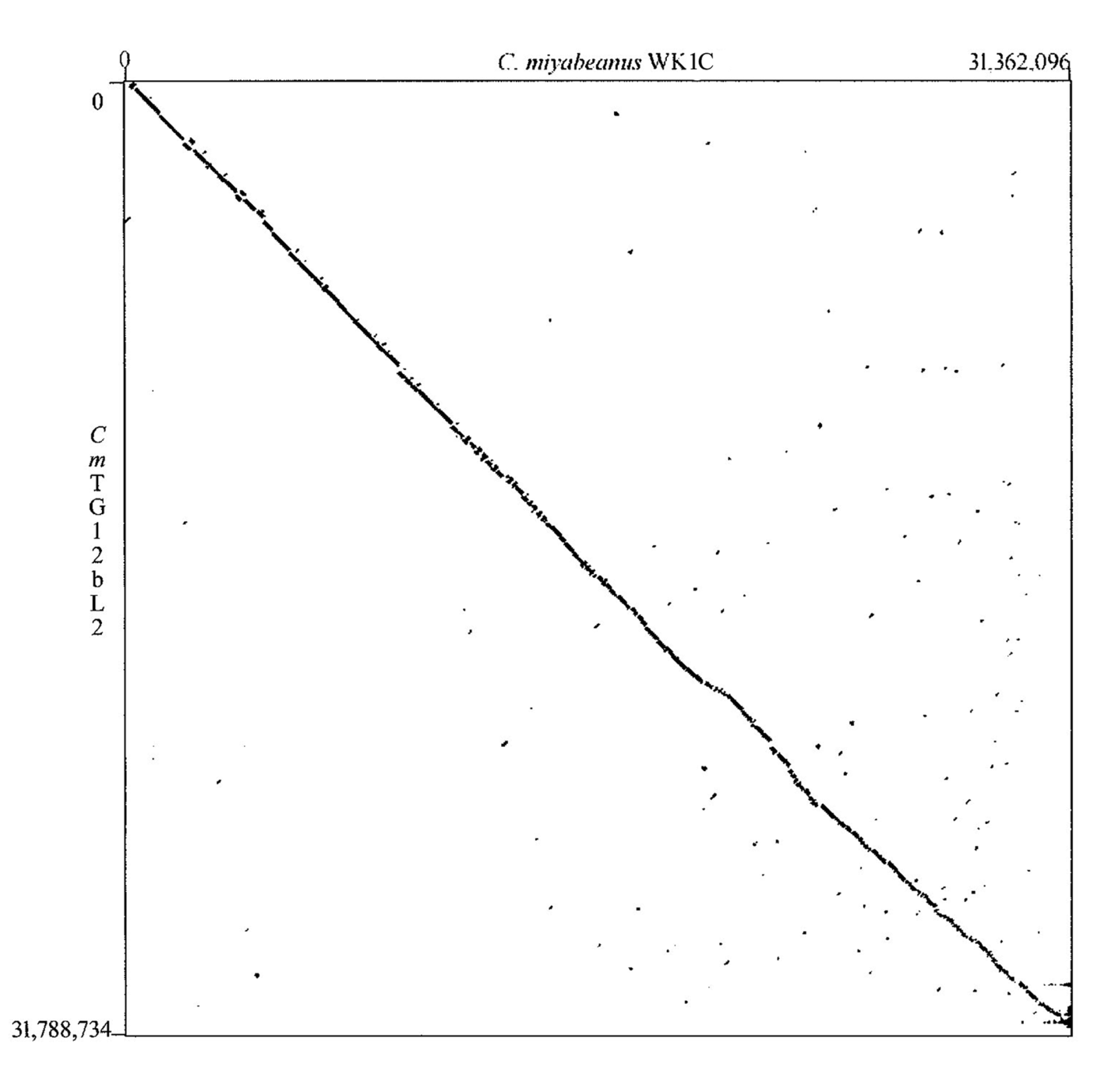

Supplement: S3 Fig — Ordered scaffolds of both species were used to build a dotplot matrix in Gepard software that uses suffix array data for heuristic dotplot computation. Minimum word length = 10. (DOCX) [file pone.0154122.s003.docx]

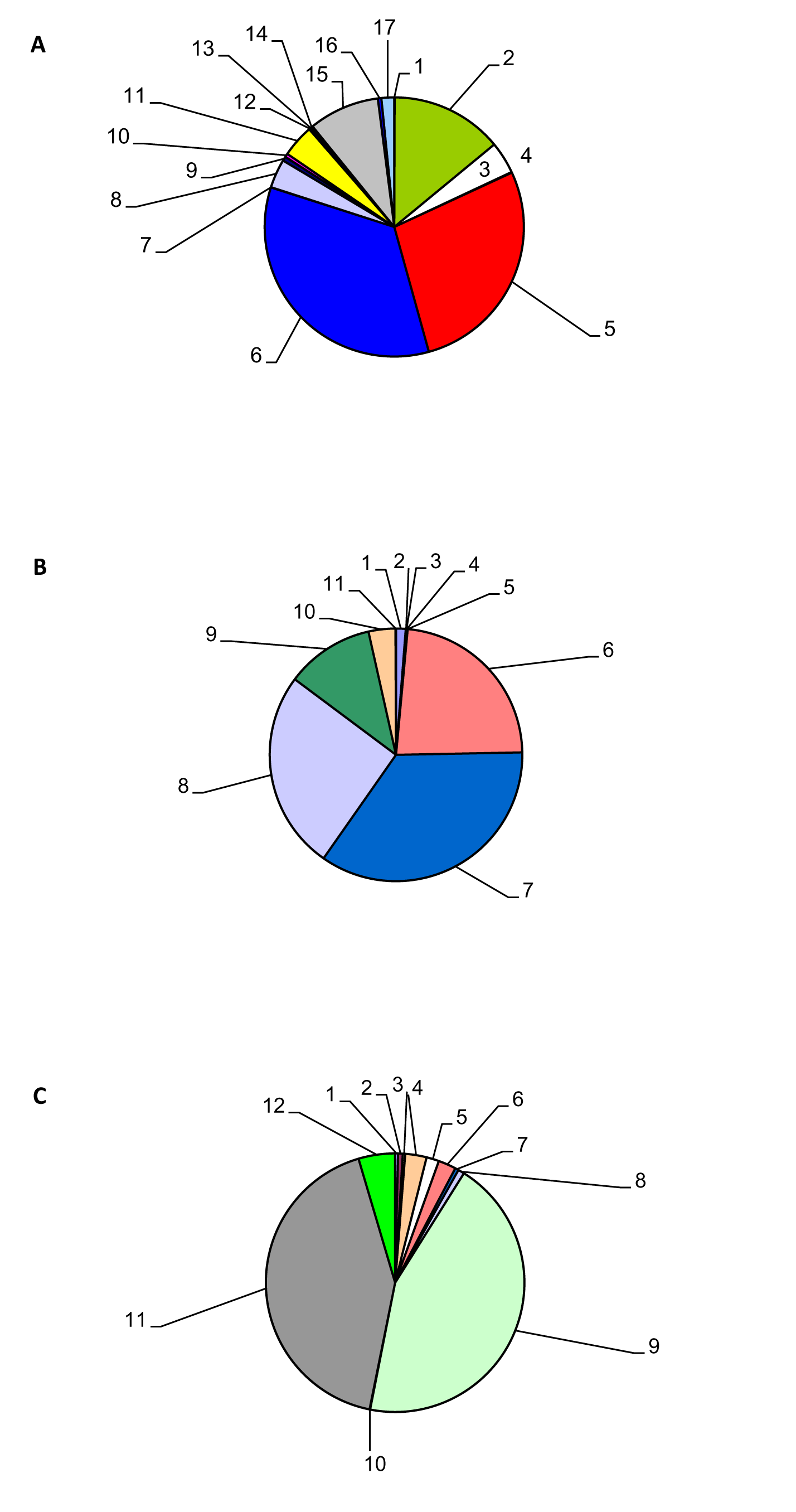

Supplement: S6 Fig — The sequence distributions by GO terms (level 2) of the fungal transcripts were done with Blast2Go software. A. Biological processes: 1. immune system process (GO:0002376), 2.single-organism process (GO:0044699), 3. response to stimulus (GO:0050896), 4. biological adhesion (GO:0022610), 5. cellular process (GO:0009987), 6. metabolic process (GO:0008152), 7. rhythmic process (GO:0048511), 8. cellular component organization or biogenesis (GO:0071840), 9. developmental process (GO:0032502), 10. reproduction (GO:0000003), 11. biological regulation (GO:0065007), 12. growth (GO:0040007), 13. locomotion (GO:0040011), 14. multi-organism process (GO:0051704), 15. localization (GO:0051179), 16. multicellular organismal process (GO:0032501), 17. signaling (GO:0023052). B. Cellular component: 1. extracellular region (GO:0005576), 2. nucleoid (GO:0009295), 3. symplast (GO:0055044), 4. synapse (GO:0045202), 5. virion (GO:0019012), 6. membrane (GO:0016020), 7. cell (GO:0005623), 8. organelle (GO:0043226), 9. macromolecular complex (GO:0032991), 10. membrane-enclosed lumen (GO:0031974), 11. cell junction (GO:0030054). C. Molecular function: 1. receptor activity (GO:0004872), 2. molecular transducer activity (GO:0060089), 3. protein binding transcription factor activity (GO:0000988), 4. nucleic acid binding transcription factor activity (GO:0001071), 5. structural molecule activity (GO:0005198), 6. electron carrier activity (GO:0009055), 7. antioxidant activity (GO:0016209), 8. enzyme regulator activity (GO:0030234), 9. binding (GO:0005488), 10. nutrient reservoir activity (GO:0045735), 11. catalytic activity (GO:0003824), 12. transporter activity (GO:0005215). (TIF) [file pone.0154122.s006.tif]

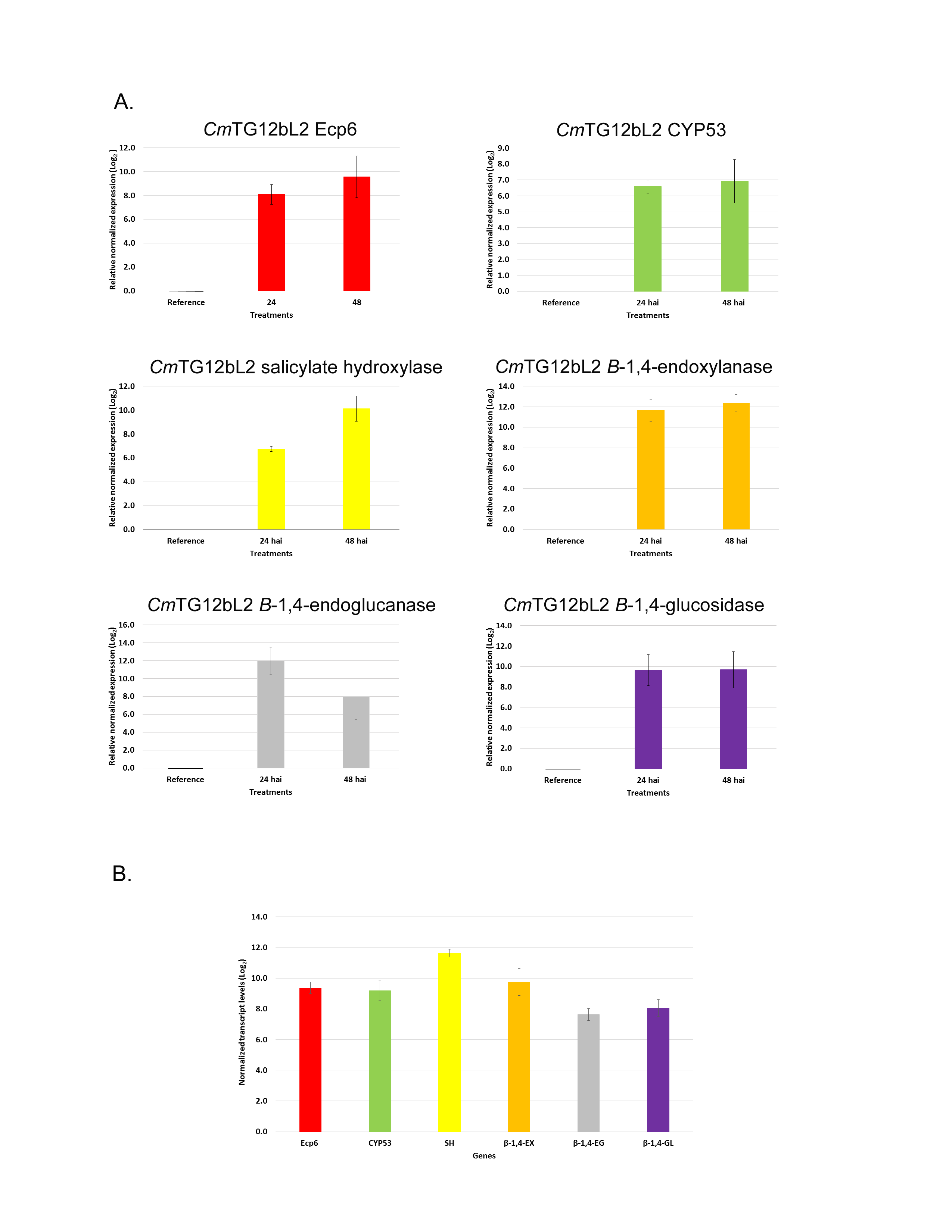

Supplement: S7 Fig — A. The vertical axis shows the log 2 of relative quantification (fold change) for each of the selected genes. The horizontal axis indicates two time points of sample collection after CmTG12Lb2 inoculation (24 h and 48 h). The reference gene expression was set to 0 (Log2 (1). Gene expression was normalized with glyceraldehyde-3-phosphate dehydrogenase expression. B. Transcript abundance (RPKM Log2 transformed values) of six genes at 48 h after inoculation. Abreviations: Ecp6 = Cladosporium fulvum Ecp6 homolog, CYP53 = Cytochrome P450 CYP53, SH = salicylate hydroxylate, β-1,4-EG = β-1,4-endoglucanase, β-1,4-EX = β-1,4-endoxylanase, β-1,4-EG = β-1,4-glucosidase. (TIF) [file pone.0154122.s007.tif]
