# Supplementary material for: Genome Assembly of the Fungus Cochliobolus miyabeanus, and Transcriptome Analysis during Early Stages of Infection on American Wildrice (Zizania palustris L.)
Source: PLoS One. 2016 Jun 2;11(6):e0154122. doi: 10.1371/journal.pone.0154122 (PMC4890743; doi:10.1371/journal.pone.0154122)
Supplement: S4 Fig — A. Multiple alignment of proteins with homology to Cf_Ecp2 = Cladosporium fulvum, Ch = C. heterostrophus; COCHEDRAFT_1130950_(gi|452001939), Cs = C. sativus; COCSADRAFT_196914_(gi|451853714), Cm = C. miyabeanus TG12bL2; CM_6804, and Fg = Fusarium graminearum (XP391494). B. Pairwise alignment of the Cf_Ecp6 and CM_2799 protein from C. miyabeanus TG12Lb2. Alignments were done with ClustalW (http://www.genome.jp/tools/clustalw/) with a dynamic programming method and using a default Blosum (for proteins) matrix, with gap penalties of 10 and gap extension penalties of 0.05. Identical amino acid residues are indicated with asterisks and highlighted in light gray, cysteines are highlighted in dark gray and LysM domains according to Cf_Ecp6 are indicated by continuous lines. (DOCX) [file pone.0154122.s004.docx]

**A**

Ch MRFSASVIVASLAATAVAAPTSPAPENVLKKRN---NFCGATTFINNSSGGSP--WITDCQTMFDRIAGDGTWVVEP--QQKRIASW

Cs MRFSTPVIVASLAASAVAAPTSPAPRSMLEKRL---NYCGATTFINNSSGGSP--WIADCQTLFDRIAGDGSWFVEP--QQKRIASW

Cm_8604 MRFSVPVIVASLAATAAAAPTSPAPGSVLEKRN---NFCGATTFINNSSGGSP--WIADCQTLFDRIAGDGTWFVEG--PQKRIASW

Fg MQFNASLLTLLMATLAAAQPVSQNPSALSFEKRGQEDQCGDSTFENKSSGGSP--KVSDCQQIARNIAGGGKWTVGAGGEHHQLVQY

Cf_Ecp2 MLFNAAAAAVFAPLLVMGNVLPRNAG--NSPGS---NRCDASTFNNGQDFDIPQAPVNDCRQMVENINRDSQFSVSH-SWARPFGGY

* *... . . . . . . : *. :** *... . * : **: : .* .. : * : : :

Ch GTCEF GARSVNN--VITTIGNEDVRDLTRDSIARFAWQGRVG----------ASGIVDCGTS----GSVKVWWGLYHT--------

Cs GTCEF GARTVNN--VITTIGNEDVRDLTRDAIARFAWQGRVG----------ASGIVDCGPQ----QNVKVWWGVYHT--------

Cm_8604 ATCEF GARSLDG--TITTIGNEDVRDLVRDSIARFAWQGRVG----------ASGVVDCGSR----GGVKVWWGVYHT--------

Fg GTCAF GAQGAGSNMNAAFIGNTDIIDLINDSIRRFEWNGLVG----------ASGVMGCRSMTGLVGGVNMRWGIYHN--------

Cf_Ecp2 GDCAF NVRVIAG-WRNGLVGGADAVDLLTDSVKNFGEANKVSSKGTYNQIVSAEGEVTCDSVDRG-GQVRVQWIVASSSYNPSNDD

. * * ..: . :*. * ** *:: .* . *. *.*.: * . *.: * : .

**B**

Cm_2799 MKSTLFAAVAILAASVSAMPYKPSKDCKSPFPNISCKPLSLQNYTIVSGDTLTTIADKFGSGACNIVAANNIANPDLIFPGQLITVP

Cf_Epc6 MQSMILFAAALMGAAVNGFVLPRTDDPDCETKATDCGSTSNIKYTVVKGDTLTSIAKKFKSGICNIVSVNKLANPNLIELGATLIIP

*:* :: *.*::.*:*..: :.* .. .* . * :**:*.*****:**.** ** ****:.*::***:** * : :*

______________________________________________

Cm_2799 ANCTETIDKTSCLPNVLQPTGTQDCVKGLSVNPPTYQVIPGDTFTLIANNFDLKLDALKNANQGRFASFDAIFAGNTTIIPVCQGCS

Cf_Ecp6 ENCSN-PDNKSCVSTPAEPT--ETCVPGLPG---SYTIVSGDTLTNISQDFNITLDSLIAAN-TQIENPDAIDVGQIITVPVCPSSQ

**:: *:.**:.. :** : ** **. :* ::.***:* *:::*::.**:* ** :: . ***. *: :*** ...

______________________________________________

Cm_2799 CTNR-NYTIVSGDTFSAIAKKGGITIGQIEAANPGQLPQSLQIGQVINNP-----VCSCVA

Cf_Ecp6 CEAVGTYNIVAGDLFVDLAATYHTTIGQIKALNNNVNPSKLKVGQQIILPQDCKNVTTAVA

* .*.**:** * :* . *****:* * . *..*::** * * * :.**

_________________________________
