# Supplementary material for: Genome Assembly of the Fungus Cochliobolus miyabeanus, and Transcriptome Analysis during Early Stages of Infection on American Wildrice (Zizania palustris L.)
Source: PLoS One. 2016 Jun 2;11(6):e0154122. doi: 10.1371/journal.pone.0154122 (PMC4890743; doi:10.1371/journal.pone.0154122)
Supplement: S5 Fig — Neighbor joining analysis of adenylation domains (AMPs) sequences of NRPS proteins (Left): I. AMPs of conserved NRPS proteins across Cochliobolus species (Red); II. AMPs of NRPS proteins with less degree of conservation in Cochliobolus species (Blue). III. AMPs of NRPS proteins with discontinuous distribution in Cochliobolus species (NRPS expanded group). IV. Outgroup set: Related adenylation modules: Long Chain Fatty Acid ligases (LCFA), Acyl-CoA synthetases (Acyl-CoA-Synth), Ochratoxin synthetases (ochratoxins), Acyl-CoA ligase (CPS1). ChetC4: Cochliobolus heterostrophus C4, ChetC5v2.0: C. heterostrophus C5, Cmiya: C. miyabeanus WK-1C, and Cmwr: C. miyabeanus TG12bL2. Other sequences are described in [40]. Blow-up clusters (Center): Main clusters harboring AMP domains related to Ch_NPS11 and GliP (gliotoxin) and SirP (sirodesmin) proteins sequences and of CmTG12bL2 NPS1/NPS3/NPS13 expanded protein. Inset (Right): Graphic representation of AMP domains belonging to C. heterostrophus NPS3 and NPS13 (blue) and to C. miyabeanus NPS1/NPS3/NPS13 expanded protein (green). (PDF) [file pone.0154122.s005.pdf]

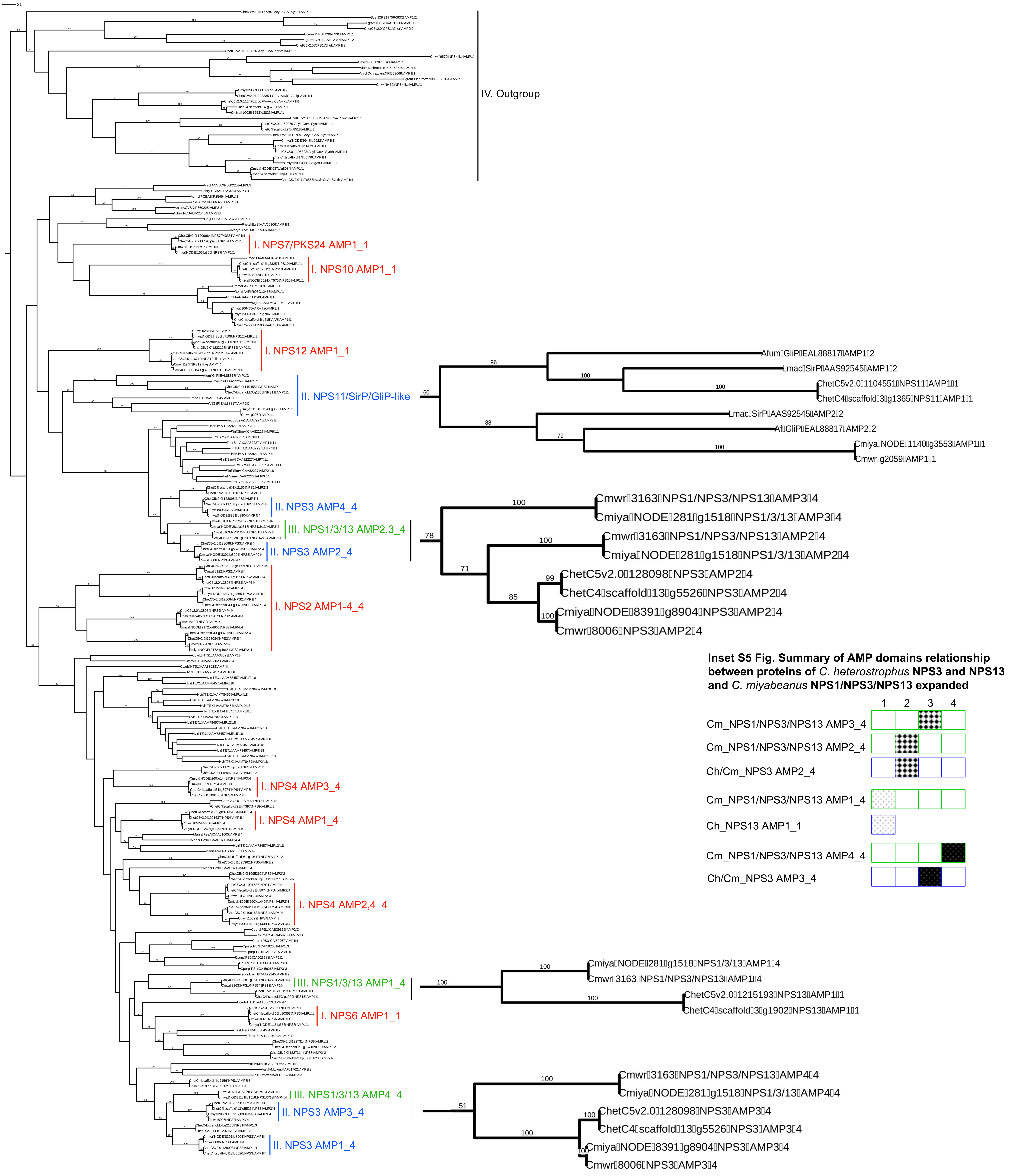
0.1
IV. Outgroup
I. NPS7/PKS24 AMP1_1
I. NPS10 AMP1_1
I. NPS12 AMP1_1
II. NPS11/SirP/GliP-like
II. NPS3 AMP4_4
III. NPS1/3/13 AMP2,3_4
II. NPS3 AMP2_4
I. NPS2 AMP1-4_4
I. NPS4 AMP3_4
I. NPS4 AMP1_4
I. NPS4 AMP2,4_4
III. NPS1/3/13 AMP1_4
I. NPS6 AMP1_1
III. NPS1/3/13 AMP4_4
II. NPS3 AMP3_4
II. NPS3 AMP1_4
Afum_GliP_EAL88817_AMP1_2
Lmac_SirP_AAS92545_AMP1_2
ChetC5v2.0_1104551_NPS11_AMP1_1
ChetC4_scaffold_3_g1365_NPS11_AMP1_1
Lmac_SirP_AAS92545_AMP2_2
Af_GliP_EAL88817_AMP2_2
Cmiya_NODE_1140_g3553_AMP1_1
Cmwr_g2059_AMP1_1
Cmwr_3163_NPS1/NPS3/NPS13_AMP3_4
Cmiya_NODE_281_g1518_NPS1/3/13_AMP3_4
Cmwr_3163_NPS1/NPS3/NPS13_AMP2_4
Cmiya_NODE_281_g1518_NPS1/3/13_AMP2_4
ChetC5v2.0_128098_NPS3_AMP2_4
ChetC4_scaffold_13_g5526_NPS3_AMP2_4
Cmiya_NODE_8391_g8904_NPS3_AMP2_4
Cmwr_8006_NPS3_AMP2_4
Inset S5 Fig. Summary of AMP domains relationship between proteins of *C. heterostrophus* NPS3 and NPS13 and *C. miyabeanus* NPS1/NPS3/NPS13 expanded
1 2 3 4
Cm_NPS1/NPS3/NPS13 AMP3_4
Cm_NPS1/NPS3/NPS13 AMP2_4
Ch/Cm_NPS3 AMP2_4
Cm_NPS1/NPS3/NPS13 AMP1_4
Ch_NPS13 AMP1_1
Cm_NPS1/NPS3/NPS13 AMP4_4
Ch/Cm_NPS3 AMP3_4
Cmiya_NODE_281_g1518_NPS1/3/13_AMP1_4
Cmwr_3163_NPS1/NPS3/NPS13_AMP1_4
ChetC5v2.0_1215193_NPS13_AMP1_1
ChetC4_scaffold_3_g1902_NPS13_AMP1_1
Cmwr_3163_NPS1/NPS3/NPS13_AMP4_4
Cmiya_NODE_281_g1518_NPS1/3/13_AMP4_4
ChetC5v2.0_128098_NPS3_AMP3_4
ChetC4_scaffold_13_g5526_NPS3_AMP3_4
Cmiya_NODE_8391_g8904_NPS3_AMP3_4
Cmwr_8006_NPS3_AMP3_4
