# Supplementary material for: Genome Assembly of the Fungus Cochliobolus miyabeanus, and Transcriptome Analysis during Early Stages of Infection on American Wildrice (Zizania palustris L.)
Source: PLoS One. 2016 Jun 2;11(6):e0154122. doi: 10.1371/journal.pone.0154122 (PMC4890743; doi:10.1371/journal.pone.0154122)
Supplement: S1 Table — a The overall % GC of all bases in the sequences followed a normal distribution overlapping the theoretical distribution. Consistently, there were no overrepresented sequences or k-mers. b Quality scores across all bases sequenced. c Percentage of duplicated sequences relative to unique sequences indicating that some sequences had 10 or more duplicates. (DOCX) [file pone.0154122.s008.docx]

| **S1 Table. Basic statistics and quality measures of the *Cochliobolus miyabeanus* TG12bL2 sequencing process**. | |
| --- | --- |
| Total (actual) number of sequences processed/single pass read | 38,531,055 |
| Sequence length (bp) | 100 |
| Overall % GC of all bases in all sequences^a^ | 50 |
| Mean sequence quality score (Phred) | 37 |
| Per base sequence quality ^b^ |  |
| Median value | 39 (1 bp) - 36 (95-99 bp) |
| Mean value | 38.5 (1bp) - 27.5 (95-99 bp) |
| Peak (range) in bp of sequence lengths distribution | 100 (99-101) |
| ‘N’ contents in any read | 0 |
| Level of duplication in category 10^c^ | ≥20 |
