# Supplementary material for: Genome Assembly of the Fungus Cochliobolus miyabeanus, and Transcriptome Analysis during Early Stages of Infection on American Wildrice (Zizania palustris L.)
Source: PLoS One. 2016 Jun 2;11(6):e0154122. doi: 10.1371/journal.pone.0154122 (PMC4890743; doi:10.1371/journal.pone.0154122)
Supplement: S1 Text — (DOCX) [file pone.0154122.s015.docx]

**CAZymes supplementary information**

The most populated group within CAZymes is GH, capable of hydrolysis and/or rearrangement of glycosidic bonds, with families GH3, GH5, GH16, GH18, GH31, GH43, and GH47 having 10 or more members each (S5 Table). Families GH3 and GH5 contain enzymes able to degrade cellulose [77], while members of the GH16 family break down β-1,3- and β-1,4-glucans that are abundant in the non-cellulose cell wall polysaccharide matrix of plants in the *Poacea* [78]. The GH43 family is enriched in cell wall degrading enzymes mostly related to hydrolysis of the arabinosyl side chains of xylan and pectin. Mostly studied in animals and plants, family GH31 contains a variety of enzymes including α-glucosidases involved in primary metabolism and breakdown of starch. GH47 are α-mannosidases involved in N-glycan processing. The GH18 family contains fungal chitinases with putative function in fungal cell wall remodeling, degradation of exogenous chitin, and defense against other organisms [79]. Some of the families contained members belonging to described subfamilies (i.e. GH5) (S5 Table), which enable more precise functional predictions.

Enzymes in the GT class catalyze the transfer of monosaccharides to diverse biological substrates yielding glycoconjugate products. The *Cm*TG12bL2 genome has a large number of GTs and the most abundant families were GT1, GT2, and GT8 with 10, 19, and nine proteins, respectively (S5 Table). Many GTs are involved in fungal cell wall biosynthesis [80]. Nine of the GT2 protein-modules identified are likely chitin synthases involved in fungal cell wall biosynthesis. Several members of the glycosyltransferase family GT1 show strong sequence similarity to sterol glucosyltransferases and could be involved in glycosylation of ergosterol and/or of plant phytoalexins. For example, one GT1 member, CM_6431, shows significant similarity to the brassinin glucosyltransferase [81].

CE catalyzes the de-O- or de*-*N*-*acetylation of substituted saccharides and demethylation of pectin chains. Families CE1, CE4, and CE5 had the most members with eight, 13, and 14 proteins, respectively (S5 Table). CAZymes of CE1 remove acetyl residues from xylan [77], a few enzymes of CE4 deacetylate chitin and derivatives [82], and members of CE5 degrade plant cuticles. Only three PL families (PL1, PL3, and PL4) with a few members each were detected in the *Cm*TG12bL2 genome. These enzymes cleave uronic acid moieties from pectic polysaccharides, which are in low abundance in cell walls of monocots.

Protein-modules of the CBM group recognize and bind carbohydrate substrates but do not have catalytic function. When tethered to a catalytic CAZyme they can increase the efficiency of polysaccharide degradation [83]. In the *Cm*TG12bL2 genome, CBMs mainly occur in families CBM1, known to bind cellulose, CBM18, which recognizes and binds chitin, and CBM50 (also known as LysM domains) that binds peptidoglycan or chitin. CBM18 was found not only as a single module but also in triplet repeats, frequently combined with GH18 (chitinase), CE4 (chitin or chitooligosaccharide deacetylase), and GH16. The *Cm*TG12bL2 genome has nine proteins containing one to seven CBM50 only domains that likely belong to the Type A classification [68] characterized by a signal peptide that are putative LysM effector proteins.

Of the three subfamilies found within AA3 (glucose-methanol-choline (GMC) oxidoreductases), AA3_2 has the most members with enzymes that generate H_2_O_2_ and assist in degrading lignin. AA9 enzymes are copper-dependent polysaccharide monooxygenases that degrade cellulose [76]. Five of them (CM_3295, CM_4151, CM_6347, CM_7519, and CM_8728) are appended to a CBM1 (Pfam00734) module, which has cellulose-binding properties. LMCOs within AA1 family could be involved in pathogenic interactions, among other roles.

Five *Cm*TG12bL2 proteins show distant relatedness to plant expansins, two of which carry a putative cellulose-binding CBM63 module. Although the role of these expansin-like proteins in fungi is uncertain, it has been proposed that they could help to loosen cell walls, and thus facilitate degradation by other enzymes [84].
